# Discovery of a new species of *Coendou* (Rodentia: Erethizontidae) within the hyper-diverse mammalian community of Sangay National Park in Ecuador

**DOI:** 10.7717/peerj.21382

**Published:** 2026-06-08

**Authors:** Jorge Brito, Jenny Curay, Víctor León-Caldas, Pamela Lojan-Cueva, Reed Ojala-Barbour, Glenda Pozo-Zamora, Laura Simba, Paul Tito, Rocío Vargas, Mateo A. Vega-Yánez, Diego Batallas

**Affiliations:** 1 Instituto Nacional de Biodiversidad, Quito, Ecuador; 2 Instituto de Diversidad y Evolución Austral (IDEAUS-CONICET), Puerto Madryn, Chubut, Argentina; 3 Parque Nacional Sangay, Macas, Ecuador; 4 Reserva de Producción de Fauna Chimborazo, Riobamba, Ecuador

**Keywords:** Eastern Andes, Cloud forest, Rare species, Biodiversity hotspot, Species delimitation

## Abstract

The tropical Andes harbor high levels of undocumented biodiversity, often hidden within complex ecological communities that require sustained sampling efforts to be fully characterized. Here, we describe a new species of porcupine of the genus *Coendou*, discovered within the hyper-diverse mammalian assemblage of Sangay National Park (Sangay) in Ecuador. The description is based on an adult specimen collected at 2,400 m on the eastern slopes of the Andes. Phylogenetic analyses using mitochondrial Cytochrome b (Cytb) place the new species as a distinct lineage within the Clade B (*sensu* (Voss, Hubbard & Jansa, 2013)), showing significant genetic divergence (p-distance > 6.0%) from its closest congeners, *C. speratus, C. nycthemera* and *C. bicolor*. Morphologically, *Coendou sangay* sp. nov. is diagnosed by its medium body size, a remarkably short tail (approx. 26% of head-and-body length), tricolored bristle-quills with brownish-red tips, and distinct cranial features, including a long nasal bone and a mesopterygoid fossa that does not reach the second upper molar. This discovery is contextualized within a comprehensive mammalian inventory of Sangay, compiled over 15 years of fieldwork. Despite an intensive sampling effort totaling 12,800 trap-nights and 2,400 camera-trap days, only a single specimen was obtained, highlighting the species status as a rare, canopy-dwelling specialist. We report 170 mammal species within the park, including 18 endemic and 35 threatened taxa. With a richness of 0.03 species per km^2^, Sangay ranks as the most mammal-diverse protected area per unit area in the Tropics. Our results demonstrate that intensive, long-term inventories are essential for identifying cryptic arboreal lineages that remain “invisible” to traditional terrestrial sampling. Finally, we emphasize the urgent need for conservation policies, including the strengthening of biological corridors and the limitation on road and mining expansion, to safeguard this high-elevation biodiversity hotspot.

## Introduction

*Coendou* Lacépède, 1799, is the most diverse genus of New World porcupines, currently comprising 22 recognized species (Voss, 2011; Menezes et al., 2021; Ramírez-Chaves et al., 2025) found in a wide range of tropical, subtropical, and Andean habitats from Mexico to Argentina (Voss, 2011; Menezes et al., 2021; Ramírez-Chaves et al., 2025). Members of this genus, commonly known as prehensile tailed porcupines, are characterized by a flexible tail that assists in climbing and arboreal locomotion (Voss, 2011). In Ecuador, four species are known to occur: *Coendou ichillus* (Voss & Da Silva, 2001) and *C. longicaudatus* Daudin, 1802 in the Amazon (Voss & Da Silva, 2001; Menezes et al., 2021), *C. rufescens* (Gray, 1865) in the Andean region (Orcés & Albuja, 2004; Voss, 2011; Narváez-Romero et al., 2018), and *C. quichua* (Thomas, 1899) in the western Andes and the coastal region (Thomas, 1899; Voss, 2011; Brito et al., 2024; Ramírez-Chaves et al., 2025).

Sangay National Park (hereafter Sangay) sits at the core of this Andean diversity, encompassing undisturbed and rugged landscapes of the Eastern Cordillera (ECOLAP & MAE, 2007). Recognized as a UNESCO World Heritage Site due to its exceptional endemism (United Nations Educational, Scientific and Cultural Organization (UNESCO), 2005), the Sangay remains one of the most logistically challenging regions for biological research in the Neotropics. The extreme topographic complexity and restricted accessibility have historically limited our understanding of its fauna, with many areas remaining virtually unexplored since early 20th-century expeditions (Moore, 1934). Few areas of Sangay are accessible by vehicle, while it is more common to traverse the rugged terrain on trails and spend the night in primitive camps. Despite these difficulties, sustained efforts have begun to unveil its mammalian complexity (Albuja et al., 1996; Lee et al., 2011; Ojala-Barbour et al., 2013; Brito & Ojala-Barbour, 2016; Lee, Tinoco & Brito, 2022). However, most inventories in these high-elevation ombrophilous forests have been temporally or spatially restricted, often failing to detect rare, low-density, or strictly canopy-dwelling lineages—taxa that are frequently underrepresented in standard terrestrial surveys.

To provide a robust baseline for this World Heritage site, we conducted a comprehensive 15-year monitoring effort (2010–2025). This inventory, which documents a total of 170 mammal species, represents one of the most intensive sampling frameworks for the Tropical Andes. It is within this extensive context of systematic sampling—and the subsequent identification of its methodological boundaries—that a unique porcupine specimen was collected. This individual, previously referred to as “*Coendou rufescens*” in preliminary reports (Brito & Ojala-Barbour, 2016; Batallas & Brito, 2022), proved to be a distinct and previously undescribed lineage.

The description of new mammalian taxa from limited material—or even singletons—is a recognized necessity in Neotropical mammalogy when dealing with rare species from inaccessible habitats (Pavan et al., 2025). By presenting the formal taxonomic description of this new *Coendou* within the results of a long-term faunal inventory, we provide the necessary ecological and sampling context to evaluate its rarity and altitudinal segregation. This integrated approach allows us to discuss why such a large mammal remained undetected despite a decade and a half of research, highlighting the “methodological invisibility” of Andean canopy fauna and the urgent conservation needs of the Sangay-Llanganates corridor.

## Materials and Methods

### Mammals inventory and sampling effort

**Figure 1:**
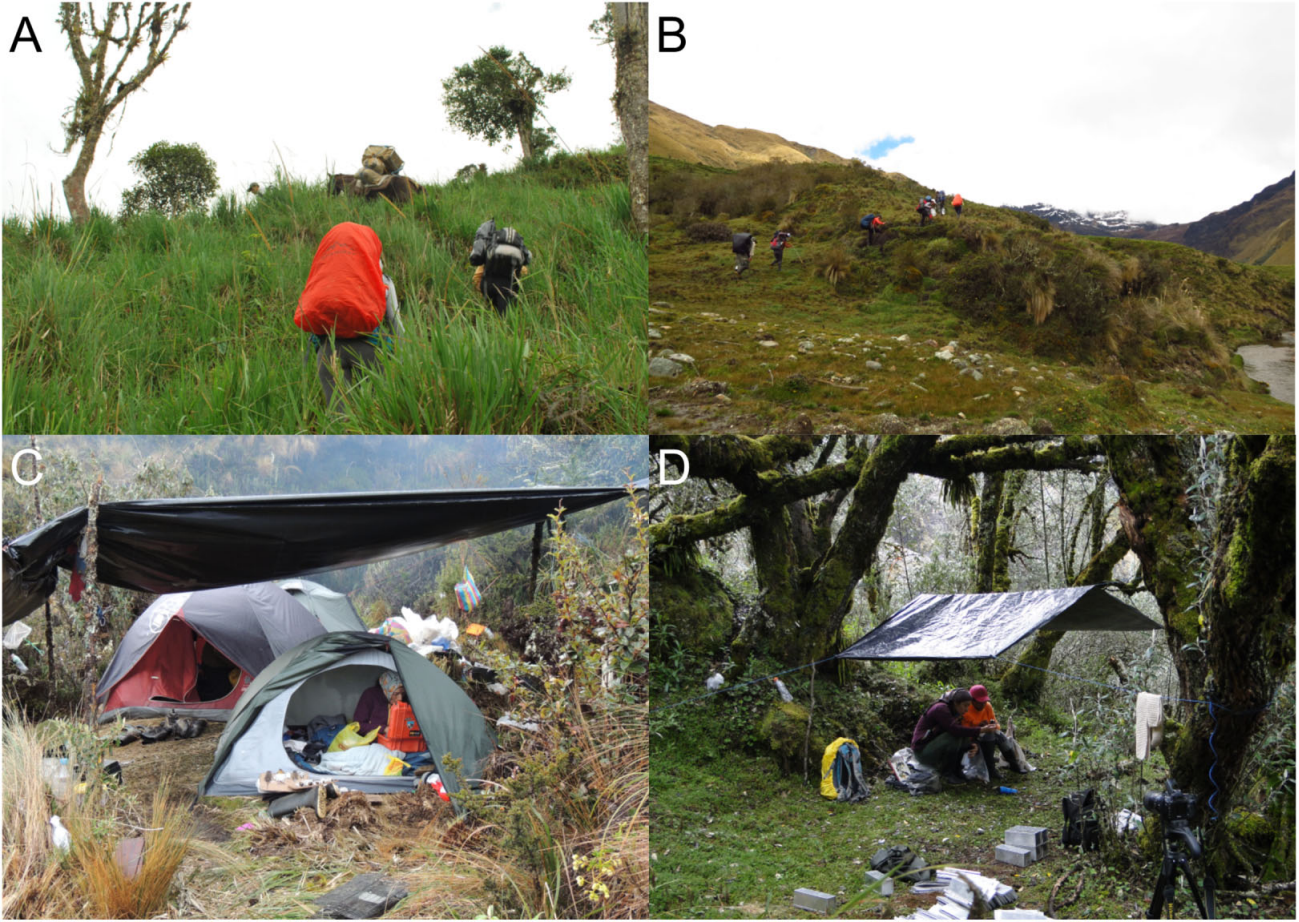
Field work. Field expedition to Guabisai (A), Cubillines (B), and sampling and collecting in the area (C, D). Photographs of J. Brito (A, C, D), and G. Pozo (B).

To establish the ecological and taxonomic context for this study, we compiled an updated list of mammals from Sangay based on long-term inventories initiated in 2010 (Fig. 1). Our field efforts involved a combination of techniques summarized by Brito & Ojala-Barbour (2016), targeting different mammalian strata across the park. Systematic sampling involved an intensive total effort of 12,800 trap-nights for non-volant small mammals (using Sherman, Tomahawk, Victor, and pitfall traps), 720 net-hours for bats, and 2,400 camera-trap nights. Camera traps were positioned primarily at ground level (0.5–1.5 m), targeting medium and large terrestrial mammals. Most specimens were obtained during multiple expeditions conducted by the senior author and collaborators (Table 1). Specimens capture, handling, and preservation procedures followed the guidelines of the American Society of Mammalogists (Sikes & Animal Care and Use Committee of the American Society of Mammalogists, 2016). Collection permits were issued by Ministerio de Ambiente y Energía de Ecuador (MAE): N° 13-2011-I-B-DPMS/MAE; 04-2012-I-B-DPMS/MAE; 005-2014-I-B-DPMS/MAE/FAUNA, and N°. MAATE-DBI-CM-2023-0334.

**Table 1:** Main sampling localities of mammals in Sangay National Park. Localities are numbered according to Fig. 2. For each site, the locality name, geographic coordinates (WGS84), elevational range (meters above sea level), and data source are provided.

N°	Location	Latitude	Longitude	Altitude	Source
1	Tungurahua	−1.44965	−78.44250	3,800–4,100	Brito et al. (2020); this study
2	Chawalyacu	−1.51011	−78.12250	1,170	Reyes-Puig, Ríos-Alvear & Bentley (2024); this study
3	Llushín	−1.59000	−78.15777	1,150	Albuja et al. (1996)
4	El Altar	−1.67159	−78.43523	3,900–4,100	Brito et al. (2020), this study
5	Cubillines	−1.76010	−78.47935	3,850–4,000	Brito et al. (2020), this study
6	Culebrillas	−1.94980	−78.49890	3,550–3,700	Brito et al. (2020)
7	Sardinayacu	−2.06838	−78.21531	1,750–2,100	Brito & Ojala-Barbour (2016); this study
8	Danu	−2.08212	−78.15498	1,350–1,500	Brito & Ojala-Barbour (2016)
9	Río Sardinayacu	−2.09632	−78.15745	1,420–1,500	Albuja et al. (1996), Brito & Ojala-Barbour (2016), Tirira et al. (2016)
10	Río Upano	−2.11695	−78.16757	1,330–1,400	Brito & Ojala-Barbour (2016)
11	Cañayacu	−2.18235	−78.20128	1,770	This study
12	Río Abanico	−2.25830	−78.20071	1,550	Albuja et al. (1996)
13	San Vicente	−2.19953	−78.36656	2,300	Brito & Ojala-Barbour (2016); this study
14	Atillo	−2.18477	−78.49645	3,400–3,600	Lee et al. (2011), Brito & Ojala-Barbour (2016)
15	Tinguichaca	−2.23580	−78.44782	2,830–2,950	Brito & Ojala-Barbour (2016)
16	Ozogoche	−2.27214	−78.59060	3,900	Albuja et al. (1996)
17	Guabisai	−2.38895	−78.30178	2,400–2,500	Brito & Ojala-Barbour (2016)
18	La Libertad	−2.63054	−78.40080	1,400–1,500	Brito & Ojala-Barbour (2016)

**Figure 2:**
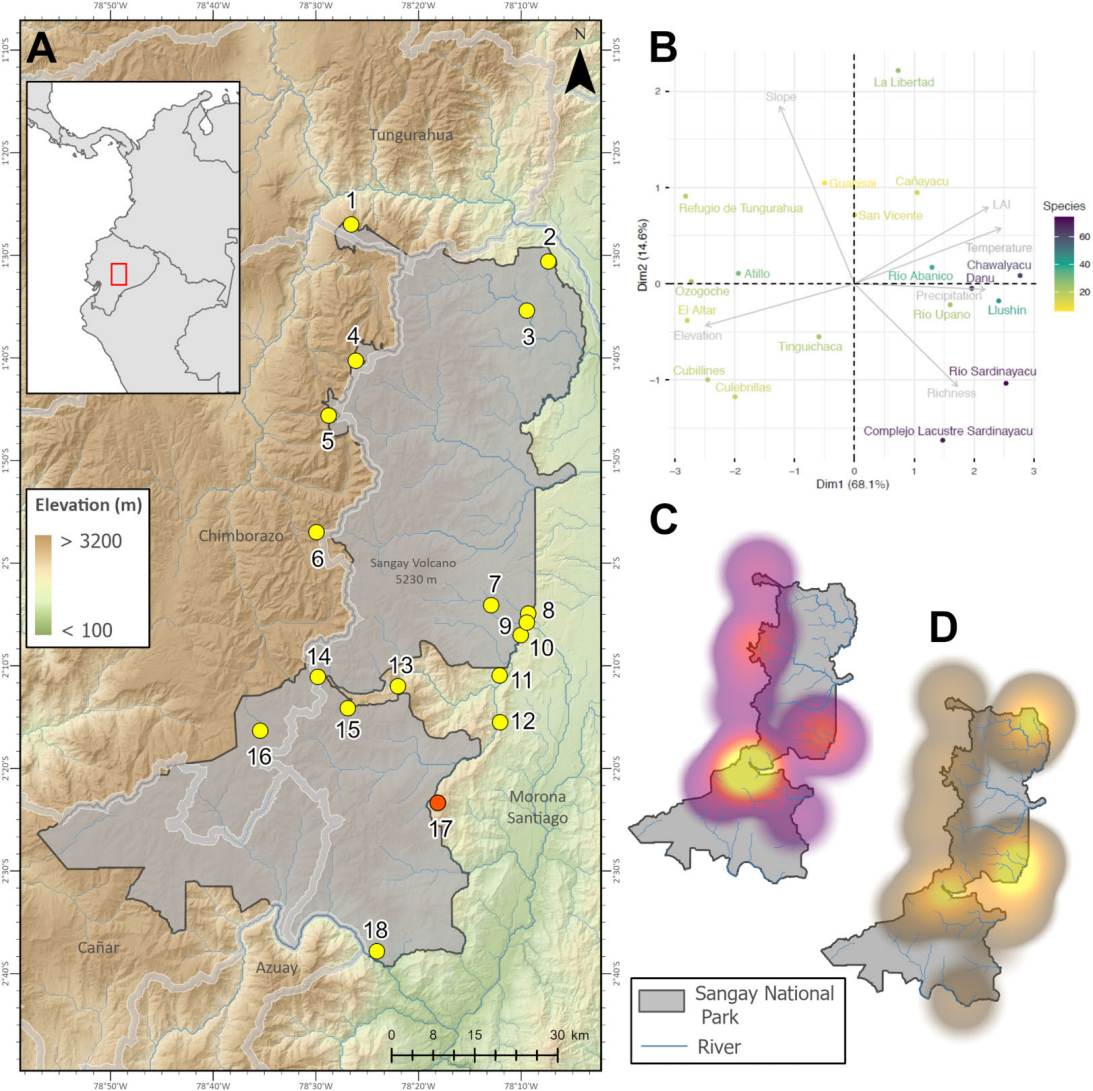
Geographic distribution, mammalian diversity patterns, and environmental variables in Sangay National Park (SNP). (A) Study area showing 18 mammal record locations; yellow dots represent sampled locations and the red dot (locality 17, Guabisai) represents the type locality of *Coendou sangay* sp. nov. (B) Principal Component Analysis (PCA) of localities; points are colored according to species richness (see scale), and vectors represent the direction and magnitude of environmental variables (elevation, temperature, precipitation, slope, and leaf area index). (C) Spatial pattern of mammalian endemism in SNP, showing concentrations in the western high-Andean zone. (D) Spatial pattern of threatened mammal species, highlighting conservation priority areas in the south-central and eastern regions of the park. Obtained from the Military Geographic Institute (IGM) of Ecuador geoportal (https://www.geoportaligm.gob.ec/portal/).

The taxonomic list was further complemented by available published information (Albuja et al., 1996; Lee et al., 2011; Brito & Ojala-Barbour, 2016; Tirira et al., 2016; Brito et al., 2020; Reyes-Puig, Ríos-Alvear & Bentley, 2024; Brito et al., 2026). The nomenclature follows Burgin et al. (2025), the global conservation status follows IUCN Standards and Petitions Committee (2024), and national conservation status follows Tirira (2021).

### Targeted collection and description of the new taxon

Despite the intensive systematic sampling described above, the discovery of the new porcupine species resulted from a targeted manual capture during an expedition in 2015. This opportunistic approach was essential to detect strictly arboreal or low-density species that are typically underrepresented or “invisible” in standard terrestrial trap-based inventories. The description is based on this single specimen (singleton; holotype) deposited at the Instituto Nacional de Biodiversidad (MECN), Quito, Ecuador. The use of a single specimen for the description of rare Neotropical mammals from inaccessible Andean regions follows established taxonomic practices when supported by clear integrative evidence (Lim, Balke & Meier, 2012; Pavan et al., 2025).

### Comparative morphological analysis

To establish a comparative framework, we examined specimens of *Coendou* spp. (File S1) from the Museo de Zoología de la Pontificia Universidad Católica del Ecuador (QCAZ), and the Escuela Politécnica Nacional (MEPN), both in Quito, Ecuador. Additionally, we reviewed high-resolution photographs of comparative material from the following international collections: Instituto Nacional de Colombia (ICN), Bogotá, Colombia; Colección de mamíferos de la Universidad del Valle (UV), Cali, Colombia; Museo de Historia Natural de la Universidad Nacional Mayor de San Marcos (MUSM), Lima, Perú, and American Museum of Natural History (AMNH), New York, NY, United States.

### Anatomy, age criteria and measurements

All measurements are in millimeters (mm), and weights are in grams (g). Head-and-body length (HBL); length of tail (LT); length of the hindfoot (HF), includes the claws; length of ear (LE). Cranial and dental measurements were taken with digital calipers to the nearest 0.1 mm following Voss & Da Silva (2001): CIL = condylo-incisive length; LD = length of diastema; MTR = maxillary toothrow length; M1-3 = length of molars; BP4 = breadth of fourth pre-molar; BM1 = breadth of first molar. APB = anterior palatal breadth; PPB = posterior palatal breadth; PZB = posterior zygomatic breadth; HIF = height of infraorbital foramen; ZL = zygomatic length; LN = length of nasals; BNA = breadth of nasal aperture; BB = breadth of braincase; DI = depth of incisor; BIT = breadth of incisor tips. The age of the specimen was classified according to the heuristic categories of Voss & Angermann (1997).

### DNA amplification and sequencing

DNA was extracted and purified from cryopreserved liver. We used GeneJET Genomic DNA Purification Kit (Thermo Fisher Scientific, Waltham, MA, USA) under the protocols of the manufacturer. We amplified a fragment of an 1,140 bp of the mitochondrial Cytochrome b (Cytb) gene *via* Polymerase Chain Reaction (PCR), using the primers MVZ05 (CTTGATATGAAAAACCATCGTTG) and MVZ14 (TCTTCATCTYHGGYTTACAAGAC) (Smith & Patton, 1993). PCR assays were conducted using a MiniPCR thermocycler machine under the following conditions: an initial denaturation at 95 °C for 120 s, followed by 35 cycles consisting of denaturation at 95 °C for 30 s, annealing at 45 °C for 30 s, and extension at 72 °C for 80 s. A final extension step was performed at 72 °C for 5 min.

### Oxford nanopore sequencing

To ensure high-quality consensus sequences, we employed Third-Generation Sequencing. A MinION Mk1C device (Oxford Nanopore Technologies, Oxford, UK) and a Flongle Flow Cells (R10.4.1) were used to sequence the samples. Library preparation was performed with the Rapid Barcoding Kit 96 (SQK-RBK114.96), according to the standard protocols of the manufacturer. To improve accuracy, the data were basecalled using the high-accuracy (HAC) model and filtered at a Qscore of 9, setting a runtime of 24 h. Consensus sequences were produced with NGSpecies ID (Sahlin, Lim & Prost, 2021). DNA extraction, PCR amplification, and sequencing were conducted at the Laboratorio de Secuenciamiento de Ácidos Nucleicos of the Instituto Nacional de Biodiversidad (INABIO) in Quito, Ecuador.

### Phylogenetic analyses

We downloaded available Cytb sequences of *Coendou* from GenBank (http://www.ncbi.nlm.nih.gov/genbank/). A DNA character matrix was constructed in Mesquite 3.81 (Maddison & Maddison, 2023), including three newly generated sequences and available congeners (File S2). The *Erethizon dorsatum* (GenBank accession: KC463889.1, FJ357428.1) was used as the outgroup. Sequences were aligned using MAFFT (Katoh & Standley, 2013) and visually inspected to corrected unambiguous alignment errors. The Cytb matrix was then partitioned by codons positions to verify reading frame continuity and the absence of premature stop codons (suggestive of NUMTs).

Phylogenetic tree were inferred using Maximum Likelihood in IQ-TREE2 (Minh et al., 2020). The best-fit substitution model for each codon partition was selected *via* ModelFinder based on the Bayesian Information Criterion (BIC). Nodal support was assessed using Ultrafast Bootstrap (UFB) with 5,000 replicates. Additionally, a Bayesian Inference (BI) was performed in BEAST v2.1 (Bouckaert et al., 2019) using a Yule tree prior and an Uncorrelated Relaxed Clock (log-normal). MCMC chains were run for 10,000,000 generations, sampling every 1,000. Convergence and stationarity were confirmed in Tracer v1.7.1 (Rambaut et al., 2018), ensuring all Effective Sample Sizes (ESS) >200. After a 15% burn-in, the Maximum Clade Credibility (MCC) tree was summarized in TreeAnnotator and visualized in FigTree v1.4.4. Uncorrected *p*-distances were calculated in MEGA 11 (Tamura, Stecher & Kumar, 2021).

### Mammal distribution patterns

To characterize the environmental patterns that structure the distribution of mammals in Sangay, a Principal Component Analysis (PCA) was applied to an integrated set of landscape, climatic, and biological variables. Topography was represented using a digital terrain model (DTM) obtained from the geoportal of the Instituto Geográfico Militar (IGM) of Ecuador (https://www.geoportaligm.gob.ec/portal/), from which slope was derived. Temperature and precipitation were used as climatic variables and extracted from WorldClim v2 (Fick & Hijmans, 2017), with a spatial resolution of 1 km^2^. Leaf Area Index (LAI) was used as an proxy for vegetation structure, calculated from MODIS (Moderate Resolution Imaging Spectroradiometer) data with a resolution of 500 m, averaging the values for the period 2000–2024 (Myneni, Knyazikhin & Park, 2015). Species richness in each location was estimated as the total number of species recorded from a presence/absence matrix and incorporated into the set of environmental variables. All variables were standardized and analyzed using PCA, implemented in R (R Core Team, 2025) with the prcomp function. The proportion of variance explained by each component was obtained from the model summary, and spatial patterns were visualized using a biplot generated with the factoextra package (Kassambara & Mundt, 2020). Locations were categorized by species richness to identify ecological gradients. Finally, spatial patterns of endemism and conservation priority were identified using kernel density heat maps generated in ArcGIS Pro based on georeferenced occurrence records.

### New zoological taxonomic names

The electronic version of this article in Portable Document Format (PDF) will represent a published work according to the International Commission on Zoological Nomenclature (ICZN), and hence the new names contained in the electronic version are effectively published under that code from the electronic edition alone. This published work and the nomenclatural acts it contains have been registered in ZooBank, the online registration system for the ICZN. The ZooBank LSIDs (Life Science Identifiers) can be resolved and the associated information viewed through any standard web browser by appending the LSID to the prefix http://zoobank.org/. The LSID for this publication is: urn:lsid:zoobank.org:pub:59B6825E-2DD2-496A-BFF1-48B47A941CAE. The online version of this work is archived and available from the following digital repositories: PeerJ, PubMed Central and CLOCKSS.

## Results

### Mammalian diversity and distribution patterns in Sangay

The long-term inventory in Sangay documented a total 170 species (representing 35.4% of Ecuador’s total mammalian diversity, *sensu* (Tirira et al., 2025)). Of these, 157 species were recorded during our 15-year fieldwork (File S2) and another 13 species have been reported in other studies for the same area. The assemblage (File S3) is dominated by rodents (54 species; 31.7%), and bats (51; 30%), followed by carnivores (22; 12.9%), didelphids opossums (11; 6.4%), peccaries (7; 4.1%), deer (8; 4.7%), primates (5; 2.9%), xenarthrans, (4; 2.3%), paucituberculates, (3; 1.7%), armadillos (2; 1.1%), tapirs (2; 1.1%), and one species each of rabbits (0.5%), and shrew (0.5%).

Notably, the Sangay serves as a sanctuary for endemic and threatened taxa, including 18 species endemic to Ecuador, five of which are currently known exclusively within the park (*Caenolestes sangay*, *Coendou sangay* sp. nov., *Rhipidomys albujai*, *Thomasomys burneoi*, and *T. salazari*), and another three (*Chilomys* sp., *Thomasomys* sp. Sangay, and *Caenolestes* sp.), could be new and are currently being evaluated. As for species threatened with extinction, 35 species are listed in a threatened category (File S3), 32 of them appear in the National Red List (Tirira, 2021) and 13 in the Global Red List (IUCN Standards and Petitions Committee, 2024).

Principal Component Analysis (PCA) revealed a clear differentiation between localities, driven primarily by an altitudinal and climatic gradient (Dim1; 68.1% of explained variance). This axis separates high-elevation, cold sites with lower species richness (*e.g*., Culebrillas, Cubillines, El Altar, and Ozogoche) from lower-elevation, warmer, and more humid localities with higher productivity (*e.g*., Chalwayaku, Danu, Río Sardinayacu, and Complejo Lacustre Sardinayacu; Figs. 2A, 2B). The second axis (Dim2; 14.6%) reflects variations in slope and topographic complexity, distinguishing sites with high environmental heterogeneity, such as Guabisai, where the new species was discovered. Spatial patterns of endemism are concentrated on the western slopes (Atillo, Tinguichaca, and Ozogoche; Fig. 2C), while threatened species are widely distributed, with a core concentration in the south-central region (Fig. 2D).

### Phylogenetic relationships and genetic divergences

**Figure 3:**
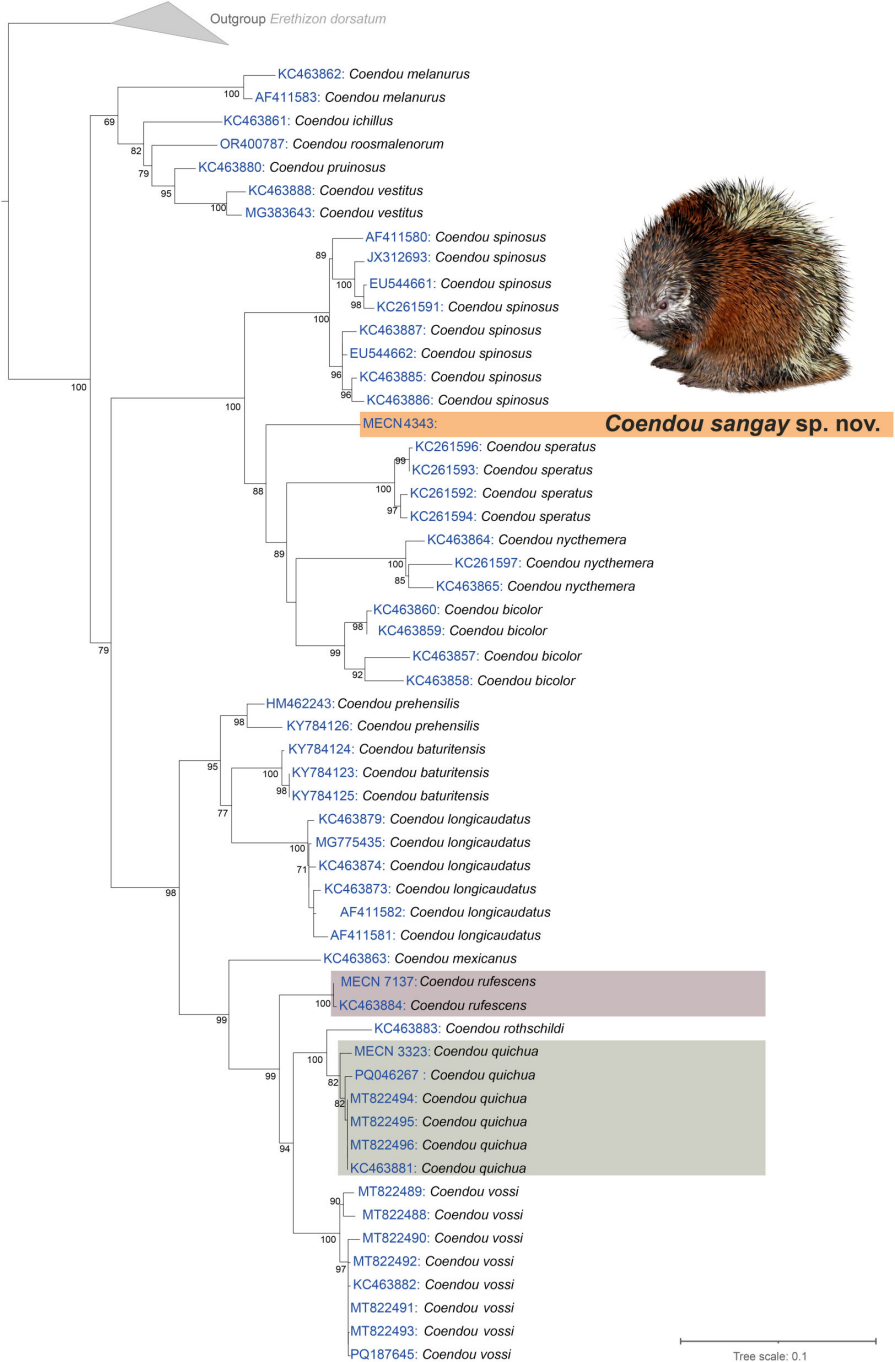
Phylogenetic relationships of *Coendou Sangay* sp. nov. Maximum Likelihood (ML) tree based on mitochondrial cytochrome b (Cytb) sequences for the genus *Coendou*. Numbers at nodes represent bootstrap support values.

The Cytb matrix comprises 55 terminals and 1,140 bp (File S3). ModelFinder in IQ-Tree2 identified the following best-fit substitution model: HKY+F+G4 for the first codon position, TN+F+G4 for the secon position, and HKY+F+I+G4 for third. Our molecular phylogeny identifies *Coendou* sp. nov. as a distinct lineage in a basal species (Fig. 3) within the clade B *sensu* (Voss, Hubbard & Jansa, 2013). It is recovered as the sister group to the cluster containing *C. spinosus*, *C. speratus*, *C. nytcthemera* and *C. bicolor* with maximum branch support (UFB = 100).

The recognition of the new species is further supported by high genetic uncorrected *p*-distance from its closest congeners within the same clade: 6.05% from *Coendou bicolor*, 6.9% from *C. speratus*, 7% from *C. spinosus* and 7.51% from *C. nycthemera* (Table 2). Furthermore, the inclusion of new sequences for *C. rufescens* and the *Coendou quichua* significantly expands the molecular dataset for Andean porcupines (Fig. 3; File S4).

**Table 2:** Genetic distances between species of *Coendou*. Uncorrected genetic *p*-distances for the mitochondrial Cytochrome b (Cytb) gene among congeners within Clade B (*sensu* Voss, Hubbard & Jansa, 2013). Values in bold indicate intraspecific variation.

	1	2	3	4	5
**1.** *Coendou* sp. nov.	*-*				
**2.** *Coendou spinosus*	7.00%	**1.88%**			
**3**. *Coendou bicolor*	6.05%	7.30%	**2.24%**		
**4**. *Coendou nycthemera*	7.51%	7.88%	6.64%	**2.1%**	
**5**. *Coendou speratus*	6.90%	7.69%	6.38%	6.92%	**0.81%**

Bayesian Inference (BI) showed that all parameters reached effective sample sizes (ESS > 200), indicating adequate MCMC convergence. Posterior probability (PP) values were high at the main nodes, supporting the monophyly of *Coendou* (PP = 1.0) and for the established clades (File S5). Bayesian analysis also places *Coendou* sp. nov. as a basal lineage to the Brazilian species (*C. speratus* and *C. nycthemera*) with moderate-to-high support (PP = 0.84). This topology is congruent with the Maximum Likelihood (ML) inference, reinforcing the consistency of its phylogenetic placement across different analytical frameworks.

### Systematic

Family Erethizontidae Bonaparte, 1845

Genus *Coendou* Lacépède, 1799

***Coendou sangay*** new species. Brito

*Coendou rufescens*: Brito & Ojala-Barbour (2016), not *Coendou rufescens* (Gray, 1865)

*Coendou rufescens*: Batallas & Brito (2022), not *Coendou rufescens* (Gray, 1865)

urn:lsid:zoobank.org:act:7E85E3A7-035F-4470-9C72-B72E98F06D5B.

Sangay Porcupine, Puerco espín de Sangay (in Spanish)

**Holotype:** MECN 4343 (field number JBM 869), an adult female collected by J. Brito, G. Pozo, J. Curay, and R. Vargas on 27 March 2015. The holotype consists of dry skin, and skull accompanied by liver, and muscle samples preserved in 95% ethanol. A complete gene sequence of Cytb from the holotype was deposited in GenBank with accession number PX654175.

**Type locality**. Ecuador, Morona Santiago province, Sucúa canton, Guabisai, Sangay National Park (−2.38895; −78.30178, WGS84 coordinates taken by GPS at the site of collection, elevation 2,400 m).

**Etymology**: This species is named in honor of Sangay National Park, which is the largest Andean national park in Ecuador. The park includes a large elevation gradient along the eastern slopes, or Eastern Cordillera, of the Andes and is recognized as a UNESCO World Heritage Site. The park gets its name from Sangay, one of Ecuador’s most active volcanoes, which is located within its boundaries.

**Diagnosis.** *Coendou sangay* sp. nov. is distinguished from other species of the genus by its medium-sized body (HBL 460 mm) and very small tail (26% LT/HBL), absence of long fur, tricolored bristle-quills (with brownish red tips), spiny ventral fur, and a unique combination of cranial features, including a long nasal bone (35% LN/CIL), constricted maxillary bony bridge, and a mesopterygoid fossa that does not reach M2.

**Figure 4:**
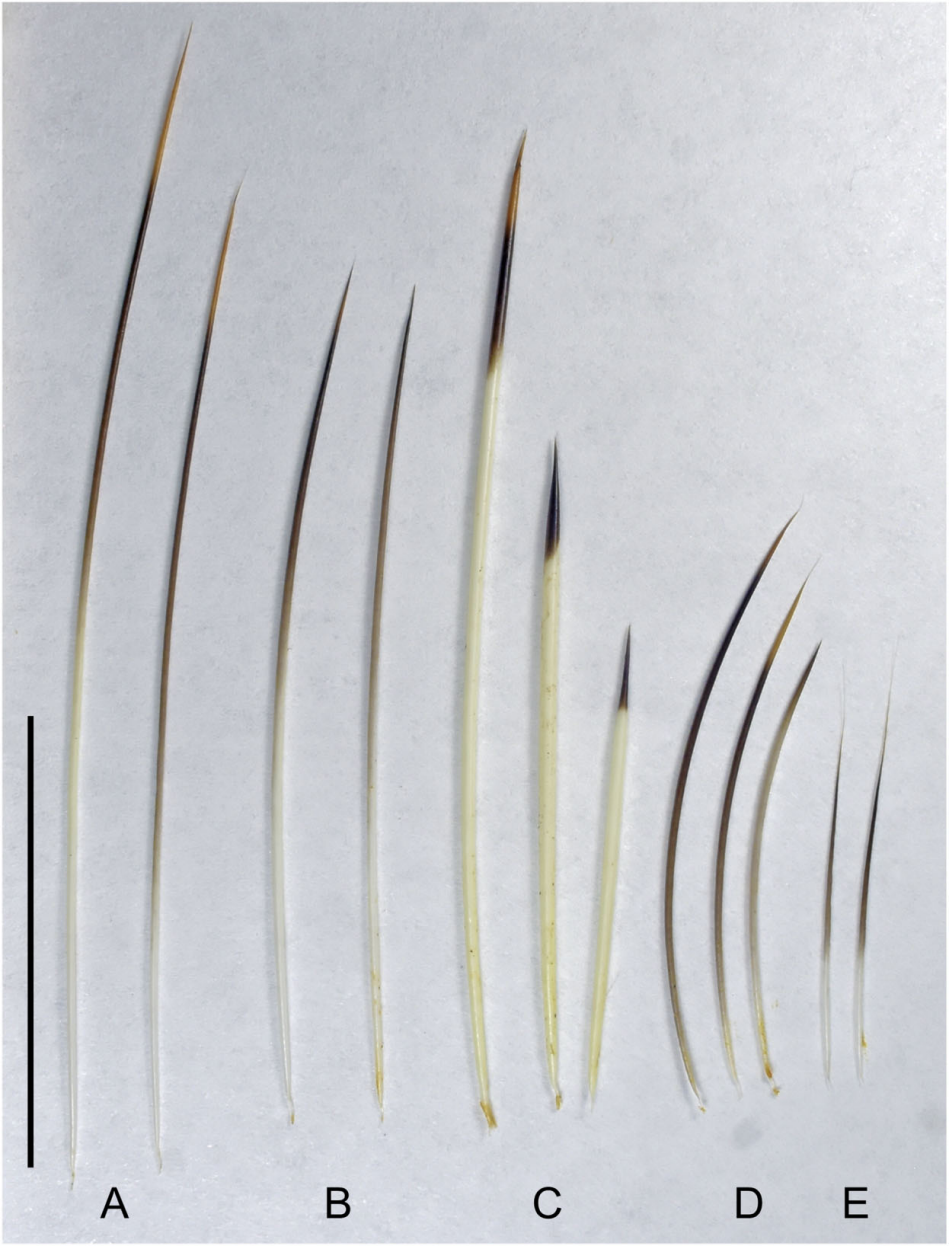
Variation in *Coendou Sangay* sp. nov. (MECN 4343, holotype) bristle-quills and quills. Mid-dorsal tricolor bristle-quills (A, B); tricolor quills from the rump (C); tricolored bristle-quills on the nape (D) and tricolored bristle-quills on the head (E). Scale = 30 mm. Photographs of J. Brito.

**Figure 5:**
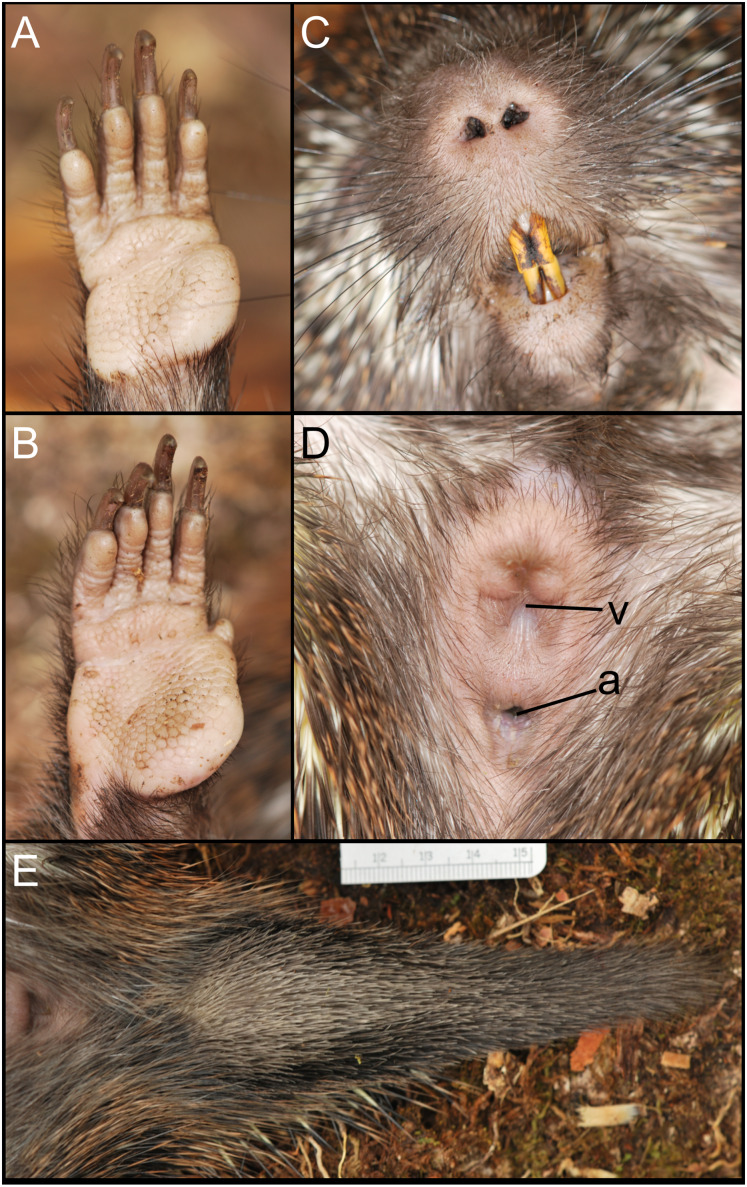
Selected external and soft anatomical features of *Coendou Sangay* sp. nov. (MECN 4343, holotype). Ventral view of the hand (A), and of the foot (B); detail of the muzzle (C); perineal region (D), and ventral view of the tail (E). Abbreviations: a = anus, v = vagina. Photographs by J. Brito.

**Figure 6:**
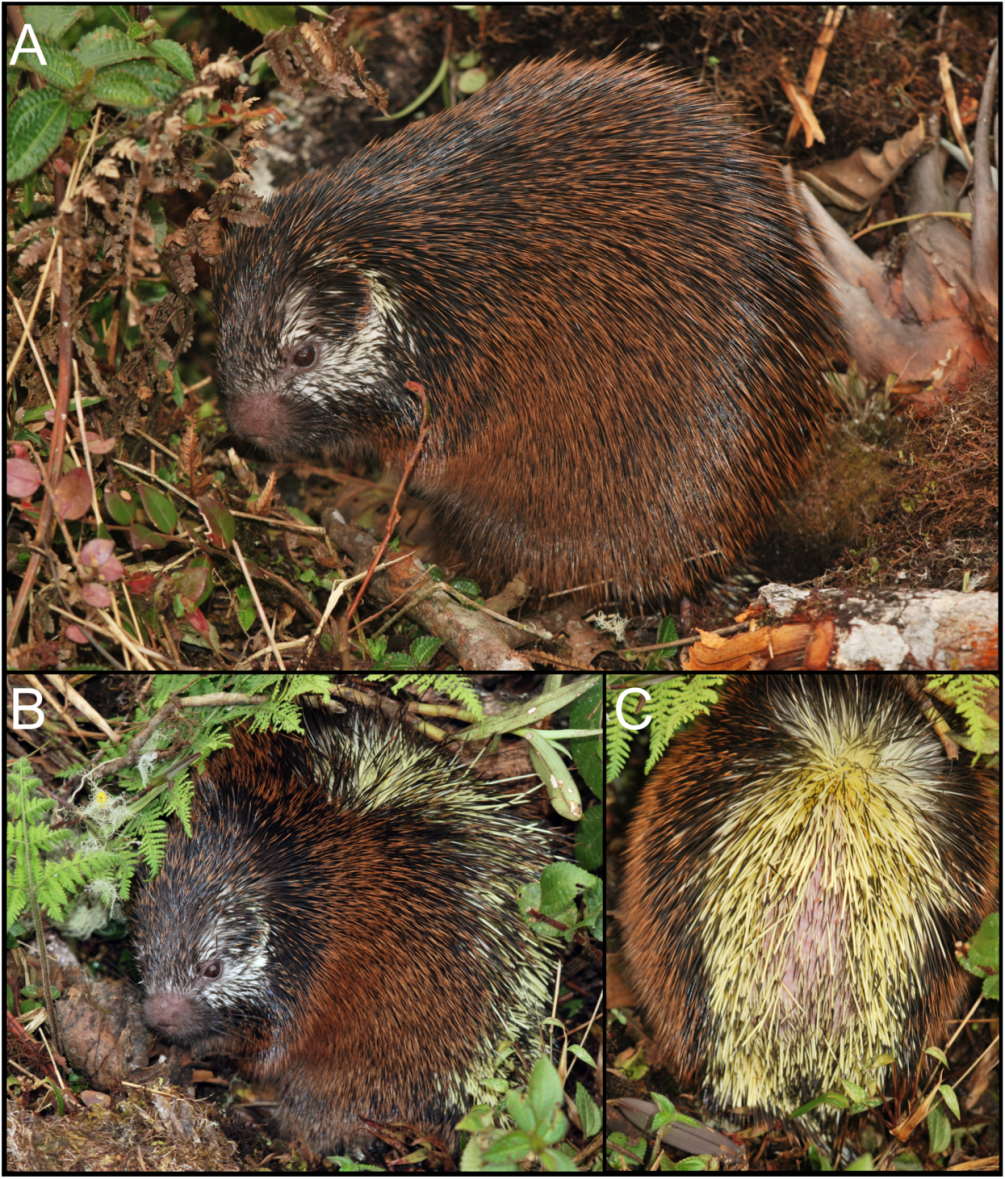
*Coendou sangay* sp. nov. (MECN 4343, holotype). (A) External appearance of the adult female alive in its natural habitat in the Sangay, Ecuador; (B) lateral and (C) posterior view of the revealing an aposematic coloration. Photographs by J. Brito.

**Morphological description of the holotype**. *Head*. Bicolored bristle-quills white basally, black in the distally (Fig. 4E); tricolored bristle-quills whitish yellow basally, brown in the middle, brownish red distally (Fig. 4D); long sparse black mystacial vibrissae, some extending beyond shoulder line (ca. 90 mm); supraocular vibrissae short (ca. 48 mm); genal vibrissae long (ca. 75 mm); pinkish bulbous muzzle covered with brownish short hairs (Figs. 5C, 6A); rounded flat ears with brown hairs on the inner side.

*Body*. The dorsal bristle-quills (Figs. 4A–4C, 6A–6C) tricolor (bright yellow basally, black in the middle, and brownish red distally) from head to mid-back, 60–95 mm-long (Figs. 4A, 4B). Quills on rump bicolor, shorter than those from head to mid-back, with larger yellow basal band, and black tip (Fig. 4C), 30–50 mm-long. Ventral surface covered with soft, 25–40 mm-long, bicolored bristle-quills, whitish basally, and reddish brown distally.

*Limbs*. The dorsal surface of fore- and hind-limbs covered with brownish dense spinous hairs; the surface is pinkish (when fresh) and densely covered with granulations (Figs. 5A, 5B). Long, curved claws present.

*Tail*. Not prehensile (Fig. 5E); dorsal surface of the proximal half covered with ca. 36 mm-long tricolored quills, similar to those on the rump, gradually shorter towards the distal half; brown spiny hairs on the lateral surface and soft brown hairs at the base of the tail; distal half of the tail covered with ca. 10 mm-long reddish brown bristles, including the ventral surface; tip of the tail with a tuft of hair 11 mm in length.

**Figure 7:**
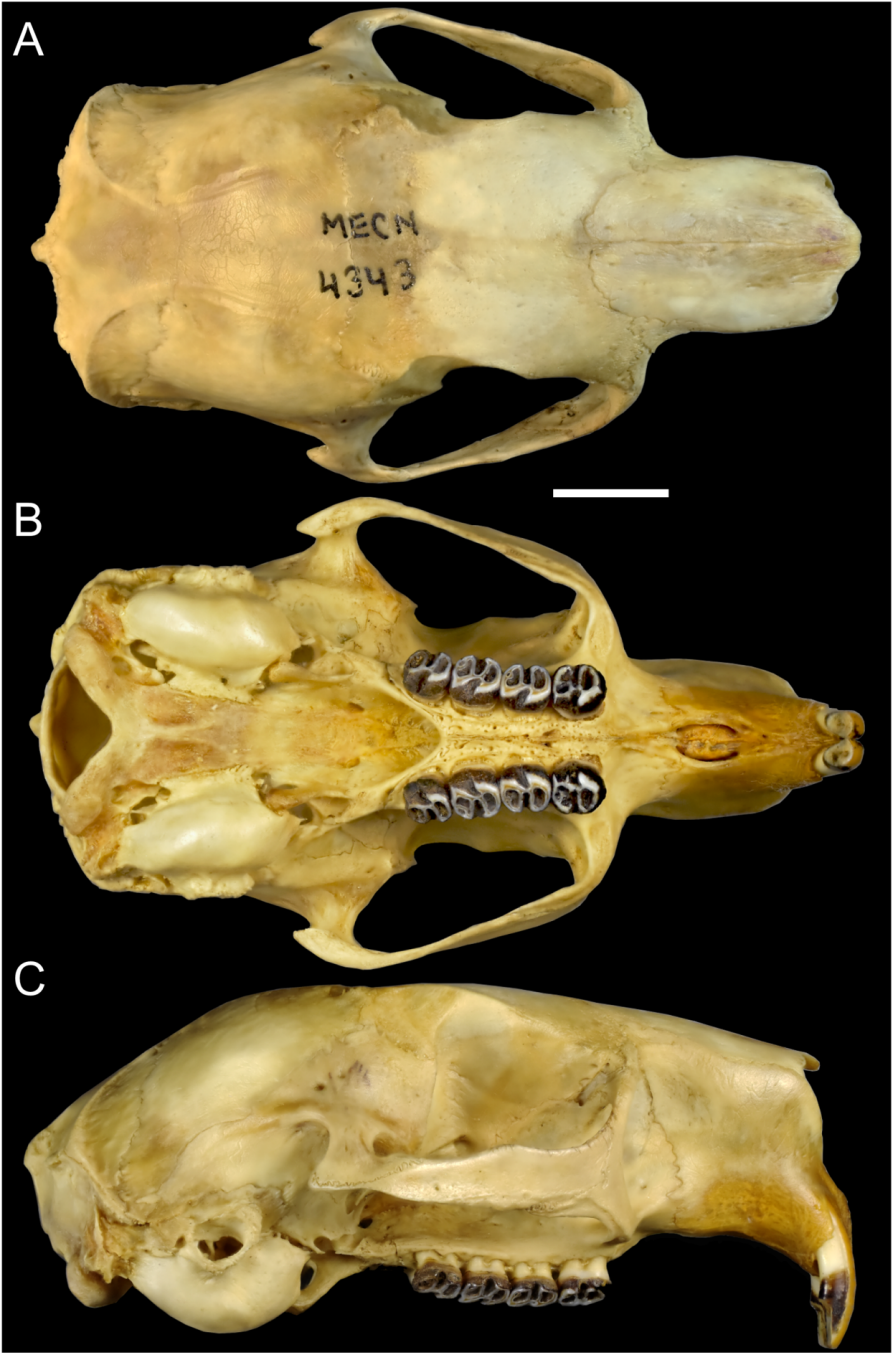
Views of the cranium. Dorsal (A), ventral (B), and lateral (C) view of the cranium of *Coendou sangay* sp. nov. (MECN 4343, holotype). Scale = 10 mm. P hotographs of J. Brito.

**Figure 8:**
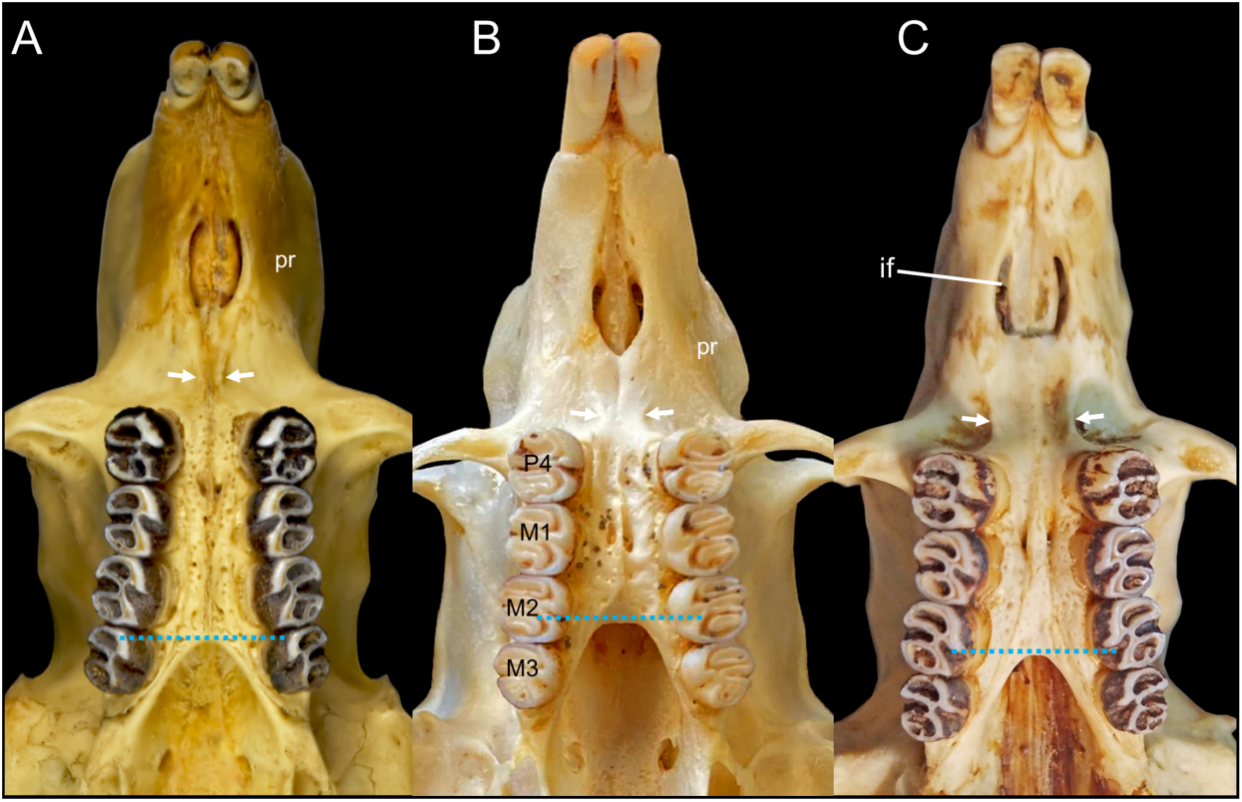
Anatomical comparisons. Constricted maxillary bone bridge (between the arrows) in *C. sangay* sp. nov. (A = MECN 4343, holotype) compared to the wider edges in *C. bicolor* (B = AMNH 147500, Ucayali, Peru) and *C. rufescens* (C = ICN 10043, Cauca, Colombia). The dotted line indicates the extent of the mesopterygoid fossa. Abbreviations: if = incisive foramen, pr = premaxillary, P4 = premolar 4, M1–M3 = molars. Photographs of J. Brito (A), R. Voss (B), and M. Botero (C).

*Skull*. Dorsal cranial profile flat over the nasals and frontals (Fig. 7A), contrasting with slightly convex surface of the parietals in lateral view (Fig. 7B); post-orbital ridges reach the protruded lambdoidal ridge; nasal bones long (35% LN/CIL), which narrow slightly backwards and forwards until they end in a point; zygomatic arches widest posteriorly, converging toward the rostrum with a slight secondary widening at the orbits; jugal expanded; right and left incisive foramina separated by a complete slim septum and bordered posteriorly by maxillary bones; constricted maxillary bony bridge (Fig. 8A); the bony palate between the toothrows is marked by a small, and median keel that is flanked by lateral gutters, these grooves extend from P4 to M3; posterior diastema and palatal bridge between cheek teeth narrowly constricted with a median keel; anterior margin of mesopterygoid fossa V-shaped, extending to the level of the third molars (Fig. 7B); roof of mesopterygoid fossa perforated by small sphenopalatine vacuities; auditory bullae large, bean-shaped, constricted posteriorly to contact paroccipital processes, leaving a portion of the petrosal bone exposed in ventral view; roof of the external auditory meatus smooth.

*Dentition*. The upper incisors have pale yellow-orange enamel bands and are moderately procumbent (Fig. 7C); maxillary toothrows weakly convergent; maxillary teeth pentalophodont, resembling those of other *Coendou* in occlusal morphology; the permanent fourth upper premolar larger than first molar, while the second molar is larger than the first and third molar.

**Holotype measurements (mm):** HBL 460, LT 120, HF 67, LE 19, W 2000, CIL 67.7, LD, 18.1, MTR 16.9, M1-3 12.5, BP4 3.9, BM1 3.8, APB 4.1, PPB 6.1, PZB 41.4, HIF 9.9, ZL 26.7, LN 23.7, BNA 11.9, BB 29.9, DI 3.1, BIT 4.7.

**Comparisons:** *Coendou sangay* sp. nov. can be easily distinguished from its sister species in clade B (*sensu* Voss, Hubbard & Jansa, 2013) by the presence of bristle-quills (absent in *C. bicolor*, *C. speratus*, and *C. nycthemera*) and the short tail, 25% LT/HBL (90–105% in *C. bicolor*, 83% in *C. speratus*, and 90% in *C. nycthemera*). Other characteristics that differentiate it from its congeners are summarized in Table 3.

**Table 3:** Diagnostic morphological and external characters of *Coendou Sangay* sp. nov. compared with closely related and geographically proximate congeners. Measurements for *C. Sangay* are based on the holotype (MECN 4343). LT/HBL, ratio of tail length to head-and-body length; MTR, maxillary toothrow length.

	*Sangay* sp. nov.	*Bicolor*	*Speratus*	*Spinosus*	*Nycthemera*	*Rufescens*
**Hindfoot (mm)**	67	76–95	57.9	60–76	60.1	62–72
**Long fur**	absent	absent	absent	present	absent	absent
**Quills**	Bicolored and tricolored	Bicolored	Bicolored and tricolored	Bicolored and tricolored	Bicolored and tricolored	Bicolored and tricolored
**Bristle-quills**	present	absent	absent	absent	absent	absent
**LT/HBL**	26%	90–105%	83%	75%	90%	35–42%
**Frontal sinuses**	Not inflated	inflated	Not inflated	Not inflated	Not inflated	Not inflated
**MTR (mm)**	17.2	19.4–21.3	14.6–16.1	15.1–17.0	14.1–16.3	16.7–19.2
**Mesopterygoid fossa**	Without reaching the M2	Reach the M2	Reach the M2	Reach the M2	Reach the M2	Without reaching the M2
**Source**	This study	Voss & Da Silva (2001)	Pontes et al. (2013)	Voss & Da Silva (2001)	Voss & Da Silva (2001)	Voss & Da Silva (2001)

**Geographic Distribution**: The new species is known only from the type locality, Guabisai, one of the mountains in Sangay National Park, at 2,400 m above sea level, in the province of Morona Santiago, Ecuador (Fig. 2).

**Figure 9:**
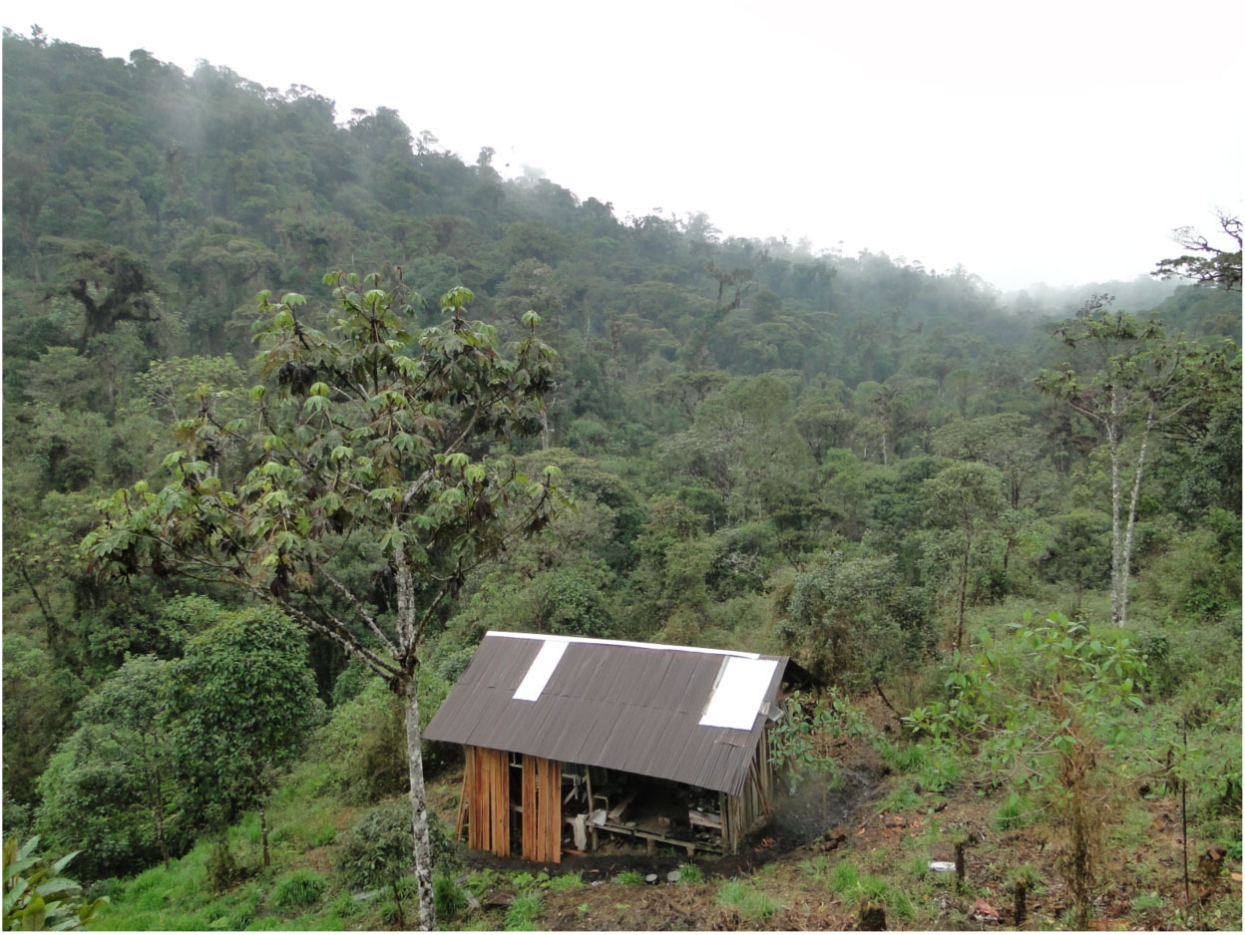
Habitat of *Coendou sangay* sp. nov. Shelter in the montane forest, the place where the holotype was collected. Photograph by J. Brito.

**Habitat and Natural history**. *Coendou sangay* sp. nov. inhabits the temperate zone (Albuja et al., 2012), in the evergreen montane forest vegetation formation of the southern Eastern Andes (Ministerio del Ambiente del Ecuador, 2013). The single observation occurred in primary cloud forest (Fig. 9), where the trees are covered with mosses and epiphytes.

On March 27, 2015, two individuals were sighted (one of them, MECN 4343, was collected manually) in the early evening as the research team approached the shelter (Fig. 9) where they were spending the night. When approached for capture, the specimen exhibited an omnidirectional erection of its quills, displaying a conspicuous aposematic coloration (Fig. 6C). With its back toward the perceived human threat, it made rapid body movements and emitted alarm calls consisting of up to four notes. The sounds varied from chirps to nasal sounds, with a dominant frequency ranging from 0.34 to 2.50 kHz (Batallas & Brito, 2022). In addition, *C. sangay* exhibited defensive mechanism where quills were easily detached upon contact, faciliting their penetration and fixation in the opponent, a behavior similar to that reported in *E. dorsatum* and *C. spinosus* (Chapman & Roze, 1997; Bessa et al., 2024). However, we did not observe the quill vibration behavior reported for *C. spinosus* (Bessa et al., 2024), nor did we record any attempted attack behavior. Marcelo Chuqui (com pers.), a local resident, told us that porcupines sporadically come to his home in groups of up to four individuals apparently looking for pieces of wood to gnaw on Brito & Ojala-Barbour (2016).

**Conservation status.** Records of *C. sangay* sp. nov. are confined exclusively to the type locality. Population size is unknown and there is limited ecological information. For these reasons, we suggest assigning the new species to the Data Deficient (DD) category, according to the IUCN Red List (2024).

## Discussion

Our phylogeny is consistent with the results published by Ramírez-Chaves et al. (2025), recovering the three lineages within the *Coendou quichua*. In our analysis, intraspecific divergence is at 2.4% between *C. quichua* sequences from Ecuador and *C. rothschildi* sequences from Panamá, 3.69% between *C. quichua* and *C. vossi* from Colombia, and 4.5% between *C. rothschildi* and *C. vossi*.

The uncorrected mean distances indicate that the newly generate *C. quichua* sequence exhibits a minimal genetic distance of 0.5% from specimen NRM 58/147399 (Ramírez-Chaves et al., 2025), and a maximum divergence of 0.87% from MECN 7959. These low values confirm their conspecificity despite the broad geographic sampling. In contrast, the uncorrected distances within the *C. rufescens* clade were remarkably low; sequences from Ecuador and Colombia differed by only 0.1%, highlighting a high degree of genetic cohesion across the northern Andes. These results underscore that the 6.05% divergence observed for *Coendou sangay* sp. nov. far exceeds the typical intraspecific variation in the genus, reinforcing its status as a distinct evolutionary lineage.

### The “singleton” challenge and sampling bias

The new species *C. sangay* sp. nov. is described from a single specimen. While the description of species based on singletons is often scrutinized due tothe limited information on intraspecific variability (Lim, Balke & Meier, 2012), it remains a necessary and valid practice in Neotropical mammalogy when supported by robust integrative evidence (Tautz et al., 2003; Padial et al., 2010; Pavan et al., 2025). In many cases, the high genetic divergence and clear phylogenetic placement provide by the Cytb marker are sufficient to validate its taxonomic identity and facilitate future comparisons (Vences et al., 2005). The main justification for describing a species from a singleton is often its rarity or the logistical challenges of its habitat (Magurran & Henderson, 2011; Pavan et al., 2025), factors that are clearly evident in the rugged terrain of Sangay.

However, the apparent scarcity of *C. sangay* sp. nov. must be interpreted within the our methodological framework. Our 15 year survey primarily used terrestrial protocols (Sherman and Tomahawk traps, pitfall traps, and ground-level camera traps), the species was never recorded. Its manual capture in the understory—and the absence of terrestrial records—highlights a significant ‘methodological invisibility’ of arboreal fauna (Voss & Emmons, 1996). Our data suggest that *C. sangay* sp. nov. may not be a low-density species in absolute terms, but rather a canopy specialist that remains undetected by traditional ground-based monitoring. This reveals a persistent ‘blind spot’ in Andean mammalogy, where even long-term terrestrial efforts are insufficient to document the complete mammalian community.

To overcome this bias in future surveys, we emphasize the transition toward multi-strata protocols. The implementation of canopy-level camera trapping (Janson & Emmons, 1990; Gregory et al., 2014) and the use of environmental DNA (eDNA) from arboreal substrates or water sources (Sales et al., 2020; Lynggaard et al., 2022) are essential to detect elusive specialists like *C. sangay* and fully characterize the biodiversity of the Tropical Andes.

### Evolutionary hypotheses for differentiation

The divergence of *C. sangay* sp. nov. within the Sangay-Llanganates corridor likely resulted from a synergy between vicariant events and niche specialization. The Eastern Cordillera of Ecuador is defined by extreme topographic complexity, shaped by rapid Andean uplift and intense Quaternary volcanic activity (Winckell, 1997; Hall et al., 2008). We hypothesize that the deep structural depressions of the Pastaza Trench (also known as the Pastaza Depression) and the subsequent uplift of adjacent Andean blocks have acted as long-term barriers to gene flow for arboreal mammals with limited dispersal capabilities (Hazzi et al., 2018). This rugged terrain creates “habitat islands” of cloud forest isolated by deep, dry valleys—a classic driver of allopatric speciation in the tropical Andes (Antonelli et al., 2009; Patterson, Solari & Velazco, 2012).

Futhermore, the type locality, Guabisai (Fig. 9), is situated within a hyper-humid montane forest characterized by constant fog and high atmospheric saturation (Fick & Hijmans, 2017; Ministerio del Ambiente del Ecuador, 2013). The morphological distinctiveness of *C. sangay*—specifically its unique tricolor bristles and exceptionally dense ventral fur—likely represents an adaptive response to these cold, water-saturated environments, enhancing thermal insulation in a high-humidity niche (Voss, 2011; Ramírez-Chaves et al., 2025). Its strictly arboreal behavior and elusive nature (Batallas & Brito, 2022) may have allowed it to persist in these “vertical refuges,” isolated from the more generalist *Coendou* lineages that occupy the western slopes or the Amazonian lowlands (Voss, Hubbard & Jansa, 2013).

### Sangay as a Neotropical hotspot

The documented richness of 170 mammal species ranks Sangay as the third most diverse protected area in the Neotropics, surpassed only by Manu National Park in Peru (222 species; Solari et al., 2006) and Yasuní National Park in Ecuador (179 species; (Tirira, Reid & Engstrom, 2018)). Furthermore, the Sangay exceed the 142 species reported for Paracou, French Guiana (Voss, Lunde & Simmons, 2001), a site traditionally considered the benchmark for intensive Neotropical mammalian inventories.

However, the geographical scale of these comparisons is critical. While Manu (17,163 km^2^) and Yasuní (10,227 km^2^) cover significantly larger areas, the Sangay comprises 5,020 km^2^. When mammalian diversity is measured per unit area, the Sangay exhibits the highest documented density with 0.03 species per km^2^, doubling or tripling the density of Yasuní (0.02) and Manu (0.01), respectively. This exceptional concentration of lineages within a compact but complex elevational gradient confirms the Sangay as a primary evolutionary laboratory. Our long-term, multi-methodological approach directly addresses the “Linnean shortfall” (Hortal et al., 2015) in the Andes, providing the necesary resolution to detect rare taxa that would otherwise remain overlooked in less intensive surveys.

### Conservation challenges

Despite its status UNESCO World Natural Heritage site, Sangay faces escalating anthropogenic pressures that threaten its unique biodiversity. Key threats identified during our long-term monitoring include: (1) habitat fragmentation driven by irregular land titling and agricultural expansion; (2) illegal hunting of large and medium-sized mammals; (3) linear infrastructure, particularly the expansion and paving of roads that bisect critical habitats; and (4) extractive activities, such as artisanal and industrial gold mining in the park’s buffer zones. These pressures are particularly concerning for habitat specialists like *C. sangay* sp. nov., whose reliance on primary cloud forest makes them highly vulnerable to forest loss. A detailed account of these threats, including specific land-tenure conflicts and their ecological implications, is provided in File S6.

### Final remarks

The exceptional mammalian diversity and endemism of in Sangay underscore the global conservation priority of this protected area. However, the escalating anthropogenic pressures—ranging from infrastructure expansion to mining—necessitate urgent, evidence-based mitigation. We propose the following strategic recommendations: (1) restricting the development of new road infrastructure within Sangay and its buffer zones to prevent further habitat fragmentation; (2) establishing a moratorium on new mining concessions in surrounding areas to safeguard the integrity of high-elevation watersheds; (3) strengthening biological corridors, specifically the Sangay–Llanganates and Sangay–Río Negro Sopladora links, to facilitate species’ elevational shifts in response to climate change; and (4) ensuring strict enforcement of land-tenure regulations to prevent further agricultural encroachment into primary forests (File S6).

The discovery of *Coendou sangay* sp. nov. within this long-term inventory highlights a critical reality in Neotropical mammalogy: even in high-diversity hotspots, rare and specialized lineages can remain undetected for decades (Lim & Engstrom, 2001; Patterson, 2002). The documentation of 170 mammal species—the highest recorded density of mammalian richness per unit area in the Tropics—is not merely a faunal list but a testament to the intensive effort required to identify this new taxon. While describing a species from a singleton is often scrutinized, our 15-year monitoring period provides the necessary ‘absence data’ to confirm that *C. sangay* is an elusive, canopy-dwelling specialist rather than a common species previously overlooked (Lim, Balke & Meier, 2012). This approach aligns with modern taxonomic standards for rare Andean taxa, where integrative evidence from limited material is essential to document hidden biodiversity (Voss, 2024; Pavan et al., 2025). Ultimately, this integrated study demonstrates that rigorous, long-term field inventories remain indispensable to addressing the Linnean and Wallacean shortfalls in the Andean-Amazonian transition (Hortal et al., 2015; Cuesta et al., 2017).

## Conclusions

The description of *Coendou sangay* sp. nov. resolves the long-standing misidentification of specimens previously assigned to *C. rufescens* and provides a crucial focal point to contextualize the broader mammalian community of Sangay. By integrating this new taxon into a comprehensive 15-year inventory, we have documented a total of 170 mammal species, revealing patterns of rarity and elevational segregation that distinguish this park as a premier global biodiversity hotspot. Our findings demonstrate that the discovery of rare, canopy-dwelling specialists is only possible through sustained, multi-methodological sampling efforts that bridge the gap between systematic monitoring and targeted taxonomic discovery. The documentation of a ‘singleton’ within such an intensive sampling framework (12,800 trap-nights and 2,400 camera-trap nights) validates the specific status of *C. sangay* as a naturally rare and elusive lineage rather than a sampling artifact. Ultimately, this work delivers both a formal taxonomic description and an ecologically robust baseline that informs urgent conservation priorities for the Sangay–Llanganates corridor, addressing the persistent Linnean and Wallacean shortfalls in one of the most topographically complex transition zones of the Tropical Andes.

## Supplemental Information

10.7717/peerj.21382/supp-1 Supplemental Information 1 Specimens examined. List of specimens of the genus *Coendou* used for morphological and taxonomic comparisons. For each record, the country, province/department, locality, and museum voucher number are provided. Institutional abbreviations follow the main text.

10.7717/peerj.21382/supp-2 Supplemental Information 2 Detailed checklist and occurrence records of mammals in Sangay National Park. Tge taxonomic classification (Order, Family, Species), precise geographic coordinates (WGS84), elevation (m a.s.l.), and sampling locality for each record. Additionally, the endemic status for Ecuador and the conservation status according to the IUCN Red List and the Red List of Mammals of Ecuador are provided.

10.7717/peerj.21382/supp-3 Supplemental Information 3 Complete taxonomic checklist of the mammals of Sangay National Park. For each species, the sampled localities (numbered according to Table 1 and Figure 2), elevational range (meters above sea level) within the park, and conservation status according to the IUCN Red List, and the Red List of Mammals of Ecuador are provided. Asterisks (*) indicate species endemic to Ecuador. This dataset represents the primary evidence for the diversity metrics discussed in the main text.

10.7717/peerj.21382/supp-4 Supplemental Information 4 Species, vouchers, and GenBank accession numbers for newly generated DNA sequences (Cytb) used in genetic analyses. The generated sequences are shown in bold.

10.7717/peerj.21382/supp-5 Supplemental Information 5 Molecular phylogeny of the genus *Coendou* based on Bayesian inference. Maximum Clade Credibility (MCC) tree illustrates the phylogenetic relationships among recognized species and the taxonomic position of Coendou sp. nov. (MECN 4343, Holotype). Numbers at nodes indicate Bayesian posterior probabilities (BPP); only values representing significant clade support are shown.

10.7717/peerj.21382/supp-6 Supplemental Information 6 Conservation threats and Implications. Extended analysis of anthropogenic threats and conservation challenges in Sangay National Park.

10.7717/peerj.21382/supp-7 Supplemental Information 7 Mitochondrial cytochrome b sequences. Sequences generated for phylogenetic analysis.

## Additional Information and Declarations

### DNA Deposition

The following information was supplied regarding the deposition of DNA sequences:

The Cytb sequences are available at GenBank: PX654175, PX654176 and PX654177.

### Data Availability

The following information was supplied regarding data availability:

The raw data is available in the Supplemental Files.

The Cytb sequences are available at GenBank: PX654175, PX654176 and PX654177.

### New Species Registration

The following information was supplied regarding the registration of a newly described species:

Publication LSID: urn:lsid:zoobank.org:pub:59B6825E-2DD2-496A-BFF1-48B47A941CAE

Coendou sangay, sp. nov. urn:lsid:zoobank.org:act:7E85E3A7-035F-4470-9C72-B72E98F06D5B;
