# Supplementary material for: Discovery of a new species of Coendou (Rodentia: Erethizontidae) within the hyper-diverse mammalian community of Sangay National Park in Ecuador
Source: PeerJ. 2026 Jun 8;14:e21382. doi: 10.7717/peerj.21382 (PMC13256124; doi:10.7717/peerj.21382)
Supplement: Supplemental Information 1 — List of specimens of the genus Coendou used for morphological and taxonomic comparisons. For each record, the country, province/department, locality, and museum voucher number are provided. Institutional abbreviations follow the main text. [file peerj-14-21382-s001.pdf]

Supplementary File S1. Specimens examined. List of specimens of the genus *Coendou* used for morphological and taxonomic comparisons. For each record, the country, province/department, locality, and museum voucher number are provided. Institutional abbreviations follow the main text.

Coendou sangay sp. nov. (*n* = 1): **Ecuador**, Morona Santiago, Parque Nacional Sangay (MECN 4343, holotype);

Coendou bicolor (*n* = 2): **Peru**, Ucayali (AMNH 147500), Cajamarca (MUSM 9398);

Coendou rufescens (*n* = 9): **Ecuador**, Azuay, Yunguilla (QCAZ 7591); Imbabura, Añaspamba (MECN 7137); **Colombia**, Cauca (ICN 10037, 10041, 10043, UV 13329, 13331,13809, 14635).

Coendou quichua (n = 6): **Ecuador**, Carchi, Reserva Drácula (MECN 7136); Santo Domingo de los Tsáchilas, La Esperanza (MECN 7360), Bosque Protector La Perla (MECN 7959); Pichincha, Púellaro (MECN 490), Cerro Campana (MECN 3025), Reserva Intillacta (MECN 8147).

Coendou ichillus (n = 1): **Ecuador**, Sucumbíos, Limoncocha (MECN 4033).

*Coendou longicaudatus (*n = 2*)*: **Ecuador**, Morona Santiago, Kutukú (MECN 3096), Zamora Chinchipe, Cordillera del Cóndor (MECN 6167).
