# Supplementary material for: Discovery of a new species of Coendou (Rodentia: Erethizontidae) within the hyper-diverse mammalian community of Sangay National Park in Ecuador
Source: PeerJ. 2026 Jun 8;14:e21382. doi: 10.7717/peerj.21382 (PMC13256124; doi:10.7717/peerj.21382)
Supplement: Supplemental Information 3 — For each species, the sampled localities (numbered according to Table 1 and Figure 2), elevational range (meters above sea level) within the park, and conservation status according to the IUCN Red List, and the Red List of Mammals of Ecuador are provided. Asterisks (*) indicate species endemic to Ecuador. This dataset represents the primary evidence for the diversity metrics discussed in the main text. [file peerj-14-21382-s003.pdf]

Supplementary File S3. Complete taxonomic checklist of the mammals of Sangay National Park. For each species, the sampled localities (numbered according to Table 1 and Figure 2), elevational range (meters above sea level) within the park, and conservation status according to the IUCN Red List, and the Red List of Mammals of Ecuador are provided. Asterisks (*) indicate species endemic to Ecuador. This dataset represents the primary evidence for the diversity metrics discussed in the main text.

**Order/Family/Species**	**Locations**	**Elevation range**	**IUCN (2024)**	**Tirira (2021)**
**Didelphimorphia**				
**Didelphidae**				
**1.** *Caluromys lanatus* (Olfers, 1818)	2, 7–8	1170–2100	LC	LC
**2.** *Chironectes minimus* (Zimmermann, 1780)	2–3, 7–9	1150–2100	LC	LC
**3.** *Didelphis marsupialis* Linnaeus, 1758	2–3 , 7–12, 18	1150–2100	LC	LC
**4.** *Didelphis pernigra* Allen, 1900	1, 4–6, 13–17	2400–4100	LC	LC
**5.** *Marmosa rubra* Tate, 1931	8	1350	DD	LC
**6.** *Marmosa waterhousei* (Tomes, 1860)	2, 8	1170–1500	NE	LC
**7.** *Marmosops caucae* (Thomas, 1900)	2, 8	1170–1500	LC	LC
**8.** *Marmosops noctivagus* (Tschudi, 1845)	2–3, 12	1170–1550	LC	DD
**9.** *Metachirus myosuros* (Temminck, 1824)	2, 9	1170–1500		LC
**10.** *Monodelphis adusta* (Thomas, 1897)	2	1170	LC	DD
**11.** *Philander andersoni* (Osgood, 1913)	2	1170	LC	LC
**Paucituberculata**				
**Caenolestidae**				
**12.** *Caenolestes fuliginosus* (Tomes, 1863)*	1, 4–6, 14, 16	3400–4100	LC	LC
**13.** *Caenolestes sangay* Ojala-Barbour, Pinto, Brito, Albuja, Lee & Patterson, 2013*	13–15	2300–3400	VU	NT
**14.** *Caenolestes* sp.	13	1300	NE	NE
**Cingulata**				
**Chlamyphoridae**				
**15.** *Cabassous unicinctus* (Linnaeus, 1758)	2–3, 9, 12	1170–1550	LC	LC
**16.** *Priodontes maximus* (Kerr, 1792)	2–3, 8	1150–1350	VU	VU
**Dasypodidae**				
**17.** *Dasypus novemcinctus* Linnaeus, 1758	2–3, 7–12, 18	1150–1770	LC	LC
**18.** *Dasypus pastasae* Krauss, 1862	2	1170	NE	DD
**Pilosa**				
**Bradypodidae**				
**19.** *Bradypus variegatus* Schinz, 1825	2–3, 10, 18	1150–1500	LC	LC
**Megalonychidae**				
**20.** *Choloepus didactylus* (Linnaeus, 1758)	2–3, 10, 12, 18	1150–1550	LC	LC
**Cyclopedidae**				
**21.** *Cyclopes ida* Thomas, 1900	10	1330	DD	LC
**Myrmecophagidae**				
**22.** *Myrmecophaga tridactyla* Linnaeus, 1758	2–3, 7	1150–2100	VU	VU
**23.** *Tamandua tetradactyla* (Linnaeus, 1758)	2–3, 7–10	1150–2100	LC	LC
**Primates**				
**Callitrichidae**				
**24.** *Leontocebus lagonotus* (Jiménez de la Espada, 1870)	2, 9	1170–1500	LC	NT
**Cebidae**				

**25.** *Cebus yuracus* Hershkovitz, 1949	2, 7, 10	1170–2100	NT	VU
**26.** *Saimiri macrodon* Elliot, 1907	2–3, 7, 10, 18	1170–2100	NE	VU
**Aotidae**				
**27.** *Aotus lemurinus* (Geoffroy St.-Hilaire, 1843)	2–3, 12	1150–1550	VU	EN
**28.** *Aotus vociferans* (Spix, 1823)	7–10, 18	1330–2100	LC	VU
**Atelidae**				
**29.** *Alouatta seniculus* (Linnaeus, 1766)	2–3, 7–8, 12	1150–2100	LC	VU
**30.** *Ateles belzebuth* Geoffroy St.-Hilaire, 1806	2	1170	EN	CR
**31.** *Lagothrix lagothricha* (Humboldt, 1812)	2, 7–8	1170–2100	EN	EN
**Rodentia**				
**Sciuridae**				
**32.** *Hadrosciurus igniventris* (Wagner, 1842)	2–3, 8–9, 12	1150–1550	LC	LC
**33.** *Microsciurus flaviventer* (Gray, 1867)	2–3, 7–10, 18	1150–2100	LC	LC
**34.** *Syntheosciurus granatensis* (Humboldt, 1811)	2–3, 7–10, 18	1150–2100	LC	LC
**Cricetidae**				
**35.** *Akodon mollis* Thomas, 1894	1, 4–6, 14–15	2830–4100	LC	LC
**36.** *Chilomys percequilloi* Brito, Tinoco, García & Pardiñas, 2022*	7	1750	NE	NE
**37**. *Chilomys* sp.	15	2830	NE	NE
**38**. *Daptomys peruviensis* (Musser & Gardner, 1974)	2	1170	LC	DD
**39**. *Euryoryzomys macconnelli* (Thomas, 1910)	2	1170	LC	LC
**40.** *Hylaeamys perenensis* (Allen, 1901)	2, 7, 8–9	1170–2100	LC	LC
**41.** *Hylaeamys tatei* (Musser, Carleton, Brothers & Gardner, 1998)*	7	1750	DD	VU
**42**. *Hylaeamys yunganus* (Thomas, 1902)	2, 7–9	1170–2100	LC	LC
**43**. *Ichthyomys orientalis* Anthony, 1923*	8	1350	NE	LC
**44**. *Microryzomys altissimus* (Osgood, 1933)	1, 4–6, 13–15	2300–4100	LC	LC
**45**. *Microryzomys minutus* (Tomes, 1860)	1, 4–6, 13–15	2300–4100	LC	LC
**46**. *Neacomys carceleni* Hershkovitz, 1940	2, 8	1170–1350	NE	LC
**47**. *Neacomys rosalindae Sánchez-Vendizú, Pacheco & Vivas-Ruiz, 2018*	2, 9	1170–1500	LC	LC
**48**. *Nectomys apicalis* Peters, 1861		1170	LC	LC
**49**. *Nephelomys auriventer* (Thomas, 1899)	7	1750	LC	LC
**50**. *Nephelomys nimbosus* (Anthony, 1926)	13, 15, 17	2300–2830	NE	LC
**51**. *Neusticomys vossi* Hanson, D'Elía, Ayers, Cox, Burneo & Lee, 2015	15	2830	NE	NT
**52**. *Oecomys bicolor* (Tomes, 1860)	2, 8, 18	1170–1500	LC	LC
**53**. *Oecomys galvez* Voss, Fleck & Giarla, 2024	2, 9	1170–1500	NE	NE
**54**. *Oligoryzomys delicatus* (Allen & Chapman, 1897)	7	1750	NE	LC
**55**. *Oreoryzomys balneator* Thomas, 1900*	7, 15	1750–2950	DD	NT
**56**. *Phyllotis haggardi* Thomas, 1908*	14	3400	LC	LC
**57.** *Rhagomys sptentrionalis* Moreno-Cárdenas, Tinoco, Albuja & Patterson, 2021*	10	1350	NE	EN

**58**. *Rhipidomys albujai* Brito, Tinoco, Chávez, Moreno-Cárdenas, Batallas & Ojala-Barbour, 2017*	7, 9	1420–2100	DD	EN
**59**. *Rhipidomys leucodactylus* (Tschudi, 1845)	2, 9	1170–1500	LC	LC
**60**. *Scolomys melanops* Anthony, 1924	3, 8	1150–1500	LC	LC
**61**. *Scolomys ucayalensis* Pacheco, 1991	9	1420	LC	LC
**62**. *Thomasomys baeops* (Thomas, 1899) *	14, 15	2830–3600	LC	LC
**63**. *Thomasomys burneoi* Lee, Tinoco & Brito, 2022*	5, 14–15	2830–3850	NE	NE
**64**. *Thomasomys cinnameus* Anthony, 1924*	15	2830	LC	NT
**65**. *Thomasomys* sp.	15	2830	NE	NE
**66**. *Thomasomys hudsoni* Anthony, 1923*	1, 14	3400–4100	VU	DD
**67**. *Thomasomys paramorum* Thomas, 1898*	4–5, 16	3850–4100	LC	LC
**68**. *Thomasomys salazari* Brito, Tinoco, Curay, Vargas, Reyes-Puig, Romero & Pardiñas, 2019*	14–15, 17	2400–3600	NE	NT
**69**. *Thomasomys taczanowskii* (Thomas, 1882)	1, 14	3400–3800	LC	NT
**Erethizontidae**				
**70**. *Coendou longicaudatus* Daudin, 1802	2–3, 10, 18	1150–1500	NE	LC
**71**. *Coendou rufescens* (Gray, 1865)	14	3400	LC	NT
**72**. *Coendou sangay* Brito, 2026*	17	2400	NE	NE
**Dinomyidae**				
**73**. *Dinomys branickii* Peters, 1873	2, 7	1170–2100	LC	EN
**Caviidae**				
**74**. *Cavia patzelti* (Schliemann, 1982) *	14, 16	3400–3900	DD	VU
**75**. *Cavia porcellus* (Linnaeus, 1758)	14	3400	NE	LC
**Dasyproctidae**				
**76**. *Dasyprocta fuliginosa* Wagler, 1832	2–3, 7–10, 12, 18	1150–2100	LC	LC
**77**. *Myoprocta pratti* Pocock, 1913	2–3	1150–1170	LC	LC
**Cuniculidae**				
**78**. *Cuniculus paca* (Linnaeus, 1766)	2–3, 7–12, 18	1150–2100	LC	NT
**79**. *Cuniculus taczanowskii* (Stolzmann, 1885)	5, 13–16	2300–3900	NT	VU
**Echimyidae**				
**80**. *Dactylomys dactylinus* (Desmarest, 1817)	8	1350	LC	LC
**81**. *Echimys saturnus* Thomas, 1928	8	1350	DD	LC
**82**. *Mesomys hispidus* (Desmarest, 1817)	2	1170	LC	LC
**83**. *Proechimys quadruplicatus* Hershkovitz, 1948	3	1150	LC	LC
**84**. *Proechimys simonsi* Thomas, 1900	7	1750	LC	LC
**Muridae**				
**85**. *Rattus rattus* Linnaeus, 1758	7	1750	LC	LC
**Lagomorpha**				
**Leporidae**				
**86**. *Sylvilagus andinus* (Thomas, 1897)	1, 4–6, 14, 16	3400–4100	DD	NT
**87**. *Sylvilagus defilippi* (Cornalia, 1850)	2–3, 12	1150–1550	NE	LC
**Eulipotyphla**				
**Soricidae**				
**88**. *Cryptotis montivagus* (Anthony, 1921)	1, 4–6, 14, 16	3400–4100	LC	NT

**Chiroptera**				
**Emballonuridae**				
**89**. *Cormura brevirostris* (Wagner, 1843)	2, 10	1150–1400	LC	LC
**90**. *Saccopteryx bilineata* (Temminck, 1838)	3	1150	LC	LC
**Phyllostomidae**				
**91**. *Desmodus rotundus (*Geoffroy St.-Hilaire, 1810)	7–9, 12	1350–2100	LC	LC
**92**. *Anoura aequatoris* Lönnberg, 1921	7–9	1350–2100	LC	NE
**93**. *Anoura caudifer* (Geoffroy St.-Hilaire, 1818)	7–9	1350–2100	LC	LC
**94**. *Anoura cultrata* Handley, 1960	7–9	1350–2100	LC	NT
**95**. *Anoura fistulata* Muchhala, Mena & Albuja, 2005	7–9	1350–2100	DD	NT
**96**. *Anoura peruana* (Tschudi, 1844)	7–9	1350–2100	LC	LC
**97**. *Micronycteris hirsuta* (Peters, 1869)	7–9	1350–2100	LC	LC
**98**. *Micronycteris megalotis* (Gray, 1842)	7–9, 12	1350–2100	LC	LC
**99**. *Micronycteris schmidtorum* (Sanborn, 1935)	7–9	1350–2100	LC	DD
**100**. *Gardnerycteris crenulatum* (Geoffroy St.-Hilaire, 1803)	7–9	1350–2100	LC	LC
**101**. *Lophostoma silvicola* d'Orbigny, 1836	7–9	1350–2100	LC	LC
**102**. *Phylloderma stenops* Peters, 1865	7a9	1350–2100	LC	LC
**103**. *Phyllostomus hastatus* (Pallas, 1767)	3, 7–9	1350–2100	LC	LC
**104**. *Tonatia maresi* Williams, Willig & Reid, 1995	3, 8–9	1350–2100	NE	LC
**105**. *Trachops cirrhosus* (Spix, 1823)	8–9	1350–2100	LC	LC
**106**. *Glyphonycteris sylvestris* Thomas, 1896	9	1420	LC	VU
**107**. *Carollia brevicauda* (Schinz, 1821)	7–9, 12	1350–2100	LC	LC
**108**. *Carollia castanea* Allen, 1890	7–9	1350–2100	LC	LC
**109**. *Carollia perspicillata* (Linnaeus, 1758)	7–9	1350–1750	LC	LC
**110**. *Rhinophylla pumilio* Peters, 1865	3, 7–9	1150–2100	LC	LC
**111**. *Sturnira bidens* Thomas, 1915	9, 14	1420–3400	LC	LC
**112**. *Sturnira bogotensis* Shamel, 1927	9, 14	1420–3400	LC	LC
**113**. *Sturnira erythromos* (Tschudi, 1844)	9, 13–16	2300–3900	LC	LC
**114**. *Sturnira giannae* Velazco & Patterson, 2019	7–9	1350–2100	NE	LC
**115**. *Sturnira magna* de la Torre, 1966	8–9	1350–1420	LC	LC
**116**. *Sturnira oporaphilum* (Tschudi, 1844)	9	1420	LC	LC
**117**. *Sturnira tildae* de la Torre, 1959	3	1150	LC	LC
**118**. *Artibeus lituratus* (Olfers, 1818)	7–9	1350–2100	LC	LC
**119**. *Artibeus obscurus* (Schinz, 1821)	7–9	1350–2100	LC	LC
**120**. *Artibeus planirostris* (Spix, 1823)	7–9	1350–2100	LC	LC
**121**. *Dermanura anderseni* (Osgood, 1916)	7–9	1350–2100	LC	LC
**122**. *Dermanura glauca* (Thomas, 1893)	7–9, 12	1350–2100	LC	LC
**123**. *Enchisthenes hartii* (Thomas, 1892)	7–9	1350–2100	LC	LC
**124**. *Mesophylla macconnelli* Thomas, 1901	7–9	1350–2100	LC	LC
**125**. *Platyrrhinus brachycephalus* (Rouk & Carter, 1972)	7–9	1350–2100	LC	LC
**126**. *Platyrrhinus helleri* (Peters, 1866)	7–9	1350–2100	LC	VU
**127**. *Platyrrhinus infuscus* (Peters, 1880)	7–9	1350–2100	LC	LC
**128**. *Platyrrhinus ismaeli* Velazco, 2005	9	1420	NT	LC
**129**. *Vampyressa melissa* Thomas, 1926	9	1420	VU	LC
**130**. *Vampyressa thyone* Thomas, 1909	8–9	1350–1550	LC	LC

**Noctilionidae**				
**131**. *Noctilio albiventris* Desmarest, 1818	3, 7	1150–2100	LC	LC
**Molossidae**				
**132**. *Molossus bondae* Allen, 1904	12	1550	LC	–
**133**. *Molossus molossus* (Pallas, 1766)	12	1550	LC	LC
**Vespertilionidae**				
**134**. *Histiotus cadenai* Rodríguez-Posada, Ramírez-Chaves & Morales-Martínez, 2021	5, 14, 16	3400–3900	NE	LC
**135**. *Histiotus humboldti* Handley, 1996	12, 13	1550–2300	DD	LC
**136**. *Neoeptesicus andinus* (Allen, 1914)	16	3900	LC	LC
**137**. *Myotis keaysi* Allen, 1914	7	1750	LC	LC
**138**. *Myotis osculatii* (Cornalia, 1849)	9	1420	LC	LC
**139**. *Myotis riparius* Handley, 1960	9	1550	LC	LC
**Carnívora**				
**Felidae**				
**140**. *Herpailurus yagouaroundi* (Geoffroy St.-Hilaire, 1803)	2, 7	1170–2100	LC	NT
**141**. *Leopardus garleppi* (Matschie, 1912)	1	3800	NT	EN
**142**. *Leopardus pardalis* (Linnaeus, 1758)	3, 7, 11–12	1150–2100	LC	NT
**143**. *Leopardus pardinoides* (Gray, 1867)	2, 7–12, 18	1170–2100	VU	VU
**144**. *Leopardus wiedii* (Schinz, 1821)	2, 10	1170–1330	NT	NT
**145**. *Puma concolor* (Linnaeus, 1771)	1–18	1150–4100	LC	EN
**146**. *Panthera onca* (Linnaeus, 1758)	2–3, 7–12, 18	1150–2100	NT	EN
**Canidae**				
**147**. *Atelocynus microtis* (Sclater, 1883)	3, 7	1150–2100	NT	VU
**148**. *Lycalopex culpaeus* (Molina, 1782)	1, 4–6, 14, 16	3400–4100	LC	VU
**149**. *Speothos venaticus* (Lund, 1842)	2–3, 7, 9	1170–2100	NT	VU
**Ursidae**				
**150**. *Tremarctos ornatus* (Cuvier, 1825)	1, 4–7, 11–17	1550–4100	VU	EN
**Procyonidae**				
**151**. *Bassaricyon alleni* Thomas, 1880	2, 8	1170–1350	LC	NT
**152**. *Nasua nasua* (Linnaeus, 1766)	2–3, 7–12, 18	1150–2100	LC	NT
**153**. *Nasua olivacea* Gray, 1865	1, 4–6, 13–16	2300–4100	NT	VU
**154**. *Potos flavus* (Schreber, 1774)	2–3, 7–12, 18	1150–2100	LC	NT
**155**. *Procyon cancrivorus* (Cuvier, 1798)	2, 7–10, 18	1170–2100	LC	LC
**Mephitidae**				
**156**. *Conepatus semistriatus* (Boddaert, 1784)	1, 4–6, 13–16	2300–4100	LC	LC
**Mustelidae**				
**157**. *Lontra longicaudis* (Olfers, 1818)	2–3, 7–10, 18	1150–2100	NT	VU
**158**. *Eira barbara* (Linnaeus, 1758)	2–3, 7, 12, 12, 18	1150–2100	LC	LC
**159**. *Galictis vittata* (Schreber, 1776)	2, 7	1170–2100	LC	DD
**160**. *Neogale africana* (Desmarest, 1818)	12	1550	LC	DD
**161**. *Neogale frenata* (Lichtenstein, 1831)	1, 5, 16	3800–3900	LC	LC
**Perissodactyla**				
**Tapiridae**				
**162**. *Tapirus pinchaque* (Roulin, 1829)	2, 6, 12–13, 15–16	1550–3550	EN	CR
**163**. *Tapirus terrestris* (Linnaeus, 1758)	2, 23, 7–9, 18	1150–2100	VU	EN
**Artiodactyla**				
**Tayassuidae**				

**164**. *Dicotyles tajacu* (Linnaeus, 1758)	2–3, 7–12, 18	1150–2100	LC	NT
**165**. *Tayassu pecari* (Link, 1795)	3	1150	VU	EN
**Cervidae**				
**166**. *Mazama americana* (Erxleben, 1777)	2–3, 6–9, 11–12, 18	1150–2100	DD	NT
**167**. *Andinocervus rufinus* (Pucheran, 1852)	1, 4–5, 11–16	1550–4100	VU	EN
**168**. *Odocoileus virginianus* (Zimmermann, 1780)	1, 4–6, 14, 16	3400–4100	LC	NT
**169**. *Passalites nemorivagus* (Cuvier, 1817)	2, 9	1170–1420	LC	NT
**170**. *Pudella mephistophiles* (de Winton, 1896)	1, 6, 16	3500–3900	DD	EN
