# Supplementary material for: Discovery of a new species of Coendou (Rodentia: Erethizontidae) within the hyper-diverse mammalian community of Sangay National Park in Ecuador
Source: PeerJ. 2026 Jun 8;14:e21382. doi: 10.7717/peerj.21382 (PMC13256124; doi:10.7717/peerj.21382)
Supplement: Supplemental Information 4 — The generated sequences are shown in bold. [file peerj-14-21382-s004.pdf]

Supplementary File S4. Species, vouchers, and GenBank accession numbers for newly generated DNA sequences (Cytb) used in genetic analyses. The sequences generated in this work are shown in bold.

Species	Voucher	GenBank	Source
C. melanurus	CRB631	AF411583.1	*Bonvicino et al., 2002*
	MNHN1997.641	KC463862.1	*Voss et al., 2013*
C. ichillus	TTU115491	KC463861.1	*Voss et al., 2013*
C. roosmalenorum	JP178	OR400787.1	*Menezes et al., 2023*
C. pruinosus	MHNLS7692	KC463880.1	*Voss et al., 2013*
C. vestitus	IAvH 7956	MG383643.1	*Ramírez-Chaves et al., 2019*
C. vestitus	AMNH 70596	KC463888.1	*Voss et al., 2013*
C. sangay **sp. nov.**	MECN 4343	PX654175	**This study**
C. spinosus	GD 252	KC463885.1	*Voss et al., 2013*
C. spinosus	UMMZ174975	KC463886.1	*Voss et al., 2013*
C. spinosus	MN46937	AF411580.1	*Bonvicino et al., 2002*
C. spinosus	UFES136	KC261591.1	*Pontes et al., 2013*
C. spinosus	-	JX312693.1	*Voloch et al., 2013*
C. spinosus	CIT1326	EU544661.1	*Vilela et al., 2009*
C. spinosus	CIT2113	EU544662.1	*Vilela et al., 2009*
C. spinosus	UFMG 3043	KC463887.1	*Voss et al., 2013*
C. bicolor	FMNH 203679	KC463860.1	*Voss et al., 2013*
C. bicolor	MUSM 9398	KC463859.1	*Voss et al., 2013*
C. bicolor	AMNH 214612	KC463857.1	*Voss et al., 2013*
C. bicolor	KU 144560	KC463858.1	*Voss et al., 2013*
C. nycthemera	USNM 519692	KC463865.1	*Voss et al., 2013*
C. nycthemera	USNM 519690	KC463864.1	*Voss et al., 2013*
C. nycthemera	UFES2079	KC261597.1	*Pontes et al., 2013*
C. speratus	UFPE1709	KC261593.1	*Pontes et al., 2013*
C. speratus	UFPE1708	KC261592.1	*Pontes et al., 2013*
C. speratus	MN72046	KC261594.1	*Pontes et al., 2013*
C. speratus	UFES1184	KC261596.1	*Pontes et al., 2013*
C. prehensilis	MN 73383	HM462243.1	*Leite et al., 2011*
C. prehensilis	UFPB 9412	KY784126.1	*Menezes et al., 2021*
C. baturitensis	UFPB 9391	KY784123.1	*Menezes et al., 2021*
C. baturitensis	UFPB 9780	KY784124.1	*Menezes et al., 2021*
C. baturitensis	UFPB 9781	KY784125.1	*Menezes et al., 2021*
C. longicautatus	USNM528360	KC463879.1	*Voss et al., 2013*
C. longicautatus	212	AF411581.1	*Bonvicino et al., 2002*
C. longicautatus	AMNH262274	KC463873.1	*Voss et al., 2013*
C. longicautatus	138	AF411582.1	*Bonvicino et al., 2002*
C. longicautatus	MNHN1997.643	KC463874.1	*Voss et al., 2013*
C. longicautatus	IAvH 6786	MG775435.1	*Torres-Martínez et al., 2019*
C. mexicanus	ASNHC6407	KC463863.1	*Voss et al., 2013*
C. rufescens	AMNH 181483	KC463884.1	*Voss et al., 2013*
C. rufescens	MECN 7137	PX654176	**This study**
C. rothschildi	USNM 296308	KC463883.1	*Voss et al., 2013*
C. quichua	MECN7959	PQ046267.1	*Brito et al., 2024*
C. quichua	KMH2218	KC463881.1	*Voss et al., 2013*
C. quichua	NRM 58/1473	MT822494.1	*Ramírez-Chaves et al., 2025*
C. quichua	NRM 58/1474	MT822495.1	*Ramírez-Chaves et al., 2025*
C. quichua	NRM 58/2704	MT822496.1	*Ramírez-Chaves et al., 2025*
C. quichua	MECN 8147	PX654177	**This study**
C. vossi	LACM27376	KC463882.1	*Voss et al., 2013*
C. vossi	MHN-UCa1616	MT822488.1	*Ramírez-Chaves et al., 2025*
C. vossi	UIS 945	MT822490.1	*Ramírez-Chaves et al., 2025*
C. vossi	UIS OLA24	MT822492.1	*Ramírez-Chaves et al., 2025*
C. vossi	UIS OLA23	MT822491.1	*Ramírez-Chaves et al., 2025*
C. vossi	MHN-UCa3432	PQ187645.1	*Ramírez-Chaves et al., 2025*
C. vossi	Unvouchered	MT822489.1	*Ramírez-Chaves et al., 2025*

C. vossi	ICN 2003	MT822493.1	*Ramírez-Chaves et al., 2025*

REFERENCES

Bonvicino CR, Penna-Firme V, Braggio E. 2002. Molecular and karyologic evidence of the taxonomic status of *Coendou* and *Sphiggurus* (Rodentia: Hystricognathi). *Journal of Mammalogy* **83(4)**:1071–1076. https://doi.org/10.1644/1545-1542(2002)083<1071:MAKEOT>2.0.CO;2

Brito J, Lojan P, Crespo J, Culebras J. 2024. First record of albinism in the Quichua Porcupine, *Coendou quichua* in Ecuador. *Therya Notes* **5**:223–227. https://doi.org/10.12933/therya_notes-24-176

Menezes FH, Feijó A, Fernandes-Ferreira H, da Costa IR, Cordeiro-Estrela P. 2021. Integrative systematics of Neotropical porcupines of *Coendou prehensilis* complex (Rodentia: Erethizontidae). *Journal of Zoological Systematics and Evolutionary Research* **59(8)**:2410–2439. https://doi.org/10.1111/jzs.12529

Menezes FH, Semedo TBF, Saldanha J, Garbino GST, Fernandes-Ferreira H, Cordeiro-Estrela P, da Costa IR. 2023. Phylogenetic relationships, distribution, and conservation of Roosmalens' dwarf porcupine, *Coendou roosmalenorum* Voss & da Silva, 2001 (Rodentia, Erethizontidae). *ZooKeys* **1179**:139–155. https://doi.org/10.3897/zookeys.1179.108766

Pontes ARM, Gadelha JR, Melo ER, de Sa FB, Loss AC, Junior VC, Costa LP, Leite YL. 2013. A new species of porcupine, genus *Coendou* (Rodentia: Erethizontidae) from the Atlantic forest of northeastern Brazil. *Zootaxa* **3636(3)**: 421–438. https://doi.org/10.11646/zootaxa.3636.3.2

Ramírez-Chaves HE, Mazepa GO, Morales-Martínez DM, Suárez-Castro AF, Colmenares-Pinzón JE, Pulido-Santacruz P, Noguera-Urbano EA. 2025. A review of the Quichua Porcupine *Coendou quichua* complex (Rodentia: Erethizontidae) with the description of a new species from Colombia. *Journal of Mammalogy* **106(3)**:764–781. https://doi.org/10.1093/jmammal/gyae140

Ramírez-Chaves HE, Torres-Martínez MM, Noguera-Urbano EA, Passos FC, Colmenares-Pinzón JE. 2019. State of knowledge and potential distribution of the Colombian endemic brown hairy dwarf porcupine *Coendou vestitus* (Rodentia). *Mammalian Biology* **99(1)**:1–11. https://doi.org/10.1016/j.mambio.2019.09.012

Torres-Martínez MM, Ramírez-Chaves HE, Noguera-Urbano EA, Colmenares-Pinzón JE, Passos FC, García J. 2019. On the distribution of the Brazilian porcupine *Coendou prehensilis* (Erethizontidae) in Colombia. *Mammalia* **83(3)**:290–297. https://doi.org/10.1515/mammalia-2018-0043

Vilela RV, Machado T, Ventura K, Fagundes V, de J Silva MJ, Yonenaga-Yassuda Y. 2009. The taxonomic status of the endangered thin-spined porcupine, *Chaetomys subspinosus* (Olfers, 1818), based on molecular and karyologic data. BMC Evolutionary Biology **9(1)**:29. https://doi.org/10.1186/1471-2148-9-29

Voloch CM, Vilela JF, Loss-Oliveira L, Schrago CG. 2013. Phylogeny and chronology of the major lineages of New World hystricognath rodents: insights on the biogeography of the Eocene/Oligocene arrival of mammals in South America. *BMC Research Notes* **6(1)**:160. https://doi.org/10.1186/1756-0500-6-160

Voss RS, Hubbard C, Jansa SA. 2013. Phylogenetic relationships of New World porcupines (Rodentia, Erethizontidae): implications for taxonomy, morphological evolution, and biogeography. *American Museum Novitates* **3769**:1–36. https://doi.org/10.1206/3769.2
