# Supplementary figures and images for: Discovery of a new species of Coendou (Rodentia: Erethizontidae) within the hyper-diverse mammalian community of Sangay National Park in Ecuador
Source: PeerJ. 2026 Jun 8;14:e21382. doi: 10.7717/peerj.21382 (PMC13256124; doi:10.7717/peerj.21382)

Tree scale: 0.01

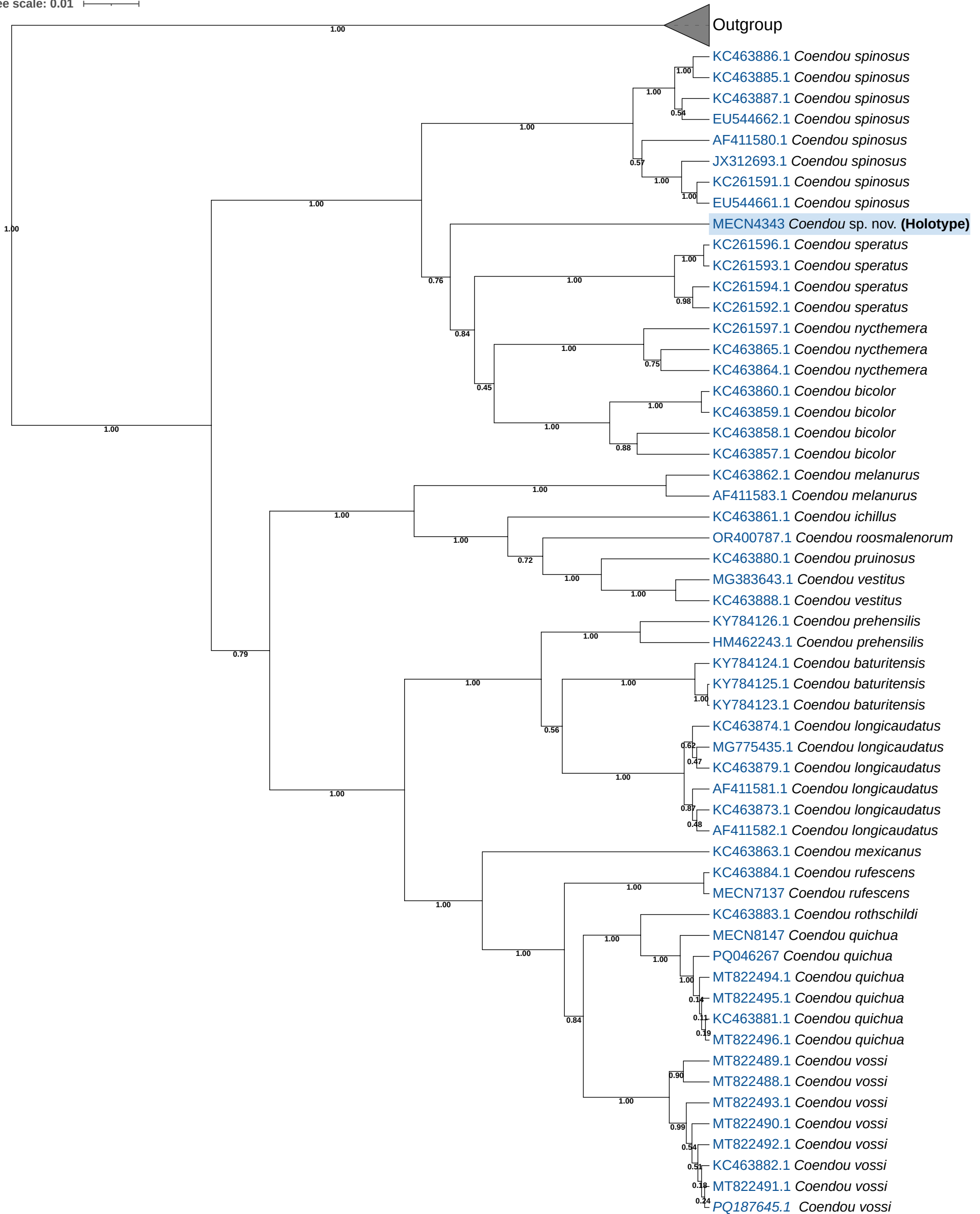

Supplement: Supplemental Information 5 — Maximum Clade Credibility (MCC) tree illustrates the phylogenetic relationships among recognized species and the taxonomic position of Coendou sp. nov. (MECN 4343, Holotype). Numbers at nodes indicate Bayesian posterior probabilities (BPP); only values representing significant clade support are shown. [file peerj-14-21382-s005.pdf]
