# Supplementary material for: Discovery of a new species of Coendou (Rodentia: Erethizontidae) within the hyper-diverse mammalian community of Sangay National Park in Ecuador
Source: PeerJ. 2026 Jun 8;14:e21382. doi: 10.7717/peerj.21382 (PMC13256124; doi:10.7717/peerj.21382)
Supplement: Supplemental Information 6 — Extended analysis of anthropogenic threats and conservation challenges in Sangay National Park. [file peerj-14-21382-s006.pdf]

Supplementary File S6. Extended analysis of anthropogenic threats and conservation challenges in Sangay National Park.

Conservation threats and Implications

Land conflicts and land claims. Sangay is home to extensive areas of primary forest and numerous areas that remain unexplored due to the inaccessibility of the terrain, suggesting the possible presence of species that have not yet been recorded (*Brito & Ojala-Barbour, 2016*). However, in 1992, its boundaries were extended southward, encompassing areas of the provinces of Morona Santiago and Cañar. This expansion left several farms with property titles and land use changes within the park in the parishes of Asunción and Sucúa (Sucúa canton), as well as in the parishes of Chinimbimi, Tayuza, and Méndez (Méndez canton). Therefore, maintaining clear boundaries between park and agricultural land uses can be difficult. These inholdings have contributed to the conversion of forest to agriculture and livestock pasture, gradually increasing pressure on ecosystems. On the other hand, in recent years, irregular property titles have emerged for large tracts of land within the protected area (ranging from 40,000 to 85,000 hectares), which appear to have been improperly registered and increase land conversion.

Hunting. Hunting pressure threatens wildlife across the park. Specifically, in parts of the Amazonian zone of the Sangay (below 1,000 m), Shuar communities traditionally practice hunting as an ancestral survival activity. However, the use of new technologies, such as firearms, has made these practices unsustainable, generating a negative impact on wildlife and ecosystems. Other poaching activity has also been observed, for example, *Albuja et al*. (*1996*) mention the existence of a mineral lick in Llushín, this place was favored by hunters to hunt medium and large mammals. In the Andean zone,

at elevations above 3,400 meters, the Indigenous communities of the Kichwa nationality of the Highlands (Puruhaes), together with mestizo populations settled in the buffer zones, selective hunting of the white-tailed deer alters the species population dynamics and negatively impacts the structure and functioning of the Andean ecosystem.

Road infrastructure. Over the years, both the central government and the municipalities of the provinces where Sangay is located have developed road infrastructure close to the park boundaries, and in some cases, such as the Macas-Riobamba highway, these roads directly cross the protected area. In addition, there is constant pressure to build an alternative road connecting Sucúa with Cañar. Similarly, the municipalities of Chambo and Pablo Sexto are seeking to open new routes linking these cities. However, the construction of these roads would pose a serious threat to the park, as it would likely increase access and land conversion in the park potentially altering its biodiversity and pristine ecosystems. In 2018 the Huangras–Riviera–Shoray road was constructed with approximately 22 km within the protected area. This intervention has generated various socio-environmental pressures associated with the surrounding communities, including Pomacocha, Juval, and Guangras, where subsistence activities are developed, leading to an increase in land-use change.

Additionally, a tertiary road was built connecting the communities of Ozogoche Alto and Ozogoche Bajo, within the Ozogoche lake complex. This project represents a potential risk of land-use transformation and could affect one of the most important wetlands in the Chimborazo province (*Echeverría, Cargua & Rosero, 2022*), due to its ecological significance and its strategic role in water supply for irrigation and human consumption.

Mining. Mining, especially gold extraction has been a historical activity in some sectors of the park, beginning with native communities and continuing during Spanish

colonization. Since 2011, the State has granted mining concessions near the boundaries of the park. (https://www.arcgis.com/apps/webappviewer/index.html?id=27bfda03ce4342b3834a27010da857e5), particularly near Palora, where mining is carried out using heavy machinery. This type of mining not only generates environmental impact (*Roy et al., 2018*), but also can lead to violence, including deaths and threats against park rangers, which makes conservation in this sector difficult (*Mestanza-Ramón et al., 2022*). As for mining activity in the park's highlands, there are currently no projects that pose a direct threat to biodiversity conservation. However, it is important to monitor mining exploration activities being carried out in the buffer zone (surrounding lands) around the park (*Palomo et al., 2013*), specifically in the parish of Pungalá, in the canton of Riobamba, in order to prevent possible future environmental impacts on the ecosystems of the protected area.

REFERENCES

Albuja L, Palacios J, Cueva M, Paredes G. (Eds). 1996. Diagnóstico faunístico para la actualización del plan de manejo del Parque Nacional Sangay. Quito, INEFAN, Proyecto GEF. 85 p.

Brito J, Ojala-Barbour R. 2016. Mamíferos no voladores del Parque Nacional Sangay, Ecuador. *Papéis Avulsos de Zoologia* **56(5):**45–61. https://doi.org/10.1590/0031-1049.2016.56.05

Echeverría M, Cargua FEC, Rosero ERR. 2022. Transitional dynamics of paramo grassland ecosystem and the primary activities through neural networks in an Andean Lake complex, Sangay National Park. *Centrosur Agraria* 1(14): 49–67. https://doi.org/10.37959/revista.v1i14.206

Mestanza-Ramón C, Cuenca-Cumbicus J, D'orio G, Flores-Toala J, Segovia-Cáceres S. Bonilla-Bonilla A, Straface S. 2022. Gold mining in the Amazon region of Ecuador: history and a review of its socio-environmental impacts. *Land* **11(2)**:221. https://doi.org/10.3390/land11020221

Palomo I, Martín-López B, Potschin M, Haines-Young R, Montes C. 2013. National Parks, buffer zones and surrounding lands: Mapping ecosystem service flows. *Ecosystem Services* **4:**104-116. https://doi.org/10.1016/j.ecoser.2012.09.001

Roy BA, Zorrilla M, Endara L, Thomas DC, Vandegrift R, Rubenstein JM, Policha T, Ríos-Touma B, Read M. 2018. New mining concessions could severely decrease biodiversity and ecosystem services in Ecuador. *Tropical Conservation Science* **11**:1940082918780427. https://doi.org/10.1177/1940082918780
